# A Comprehensive Bioinformatics Analysis of *UBE2C* in Cancers

**DOI:** 10.3390/ijms20092228

**Published:** 2019-05-07

**Authors:** Hassan Dastsooz, Matteo Cereda, Daniela Donna, Salvatore Oliviero

**Affiliations:** 1 Dipartimento di Scienze della Vita e Biologia dei Sistemi, Università di Torino, Via Accademia Albertina, 13, 10123 Torino, Italy; hassan.dastsooz@unito.it (H.D.); daniela.donna@unito.it (D.D.); 2 Italian Institute for Genomic Medicine (IIGM), Via Nizza 52, 10126 Torino, Italy; matteo.cereda@iigm.it

**Keywords:** *UBE2C*, TCGA, GEPIA, GTEx, UALCAN, overexpression, cancer

## Abstract

Ubiquitination is one of the main post-translational modification of proteins. It plays key roles in a broad range of cellular functions, including protein degradation, protein interactions, and subcellular location. In the ubiquitination system, different proteins are involved and their dysregulation can lead to various human diseases, including cancers. By using data available from the Cancer Genome Atlas (TCGA) and the Genotype-Tissue Expression (GTEx) databases, we here show that the ubiquitin conjugating enzyme, E2C (*UBE2C*), is overexpressed in all 27 cancers we investigated. *UBE2C* expression is significantly higher in late-stage tumors, which might indicate its involvement in tumor progression and invasion. This study also revealed that patients with higher *UBE2C* levels showed a shorter overall survival (OS) time and worse OS prognosis. Moreover, our data show that *UBE2C* higher-expression leads to worse disease-free survival prognosis (DFS), indicating that *UBE2C* overexpression correlates with poor clinical outcomes. We also identified genes with positive correlations with *UBE2C* in several cancers. We found a number of poorly studied genes (family with sequence similarity 72-member D, *FAM72D*; meiotic nuclear divisions 1, *MND1*; mitochondrial fission regulator 2, *MTFR2*; and POC1 centriolar protein A, *POC1A*) whose expression correlates with *UBE2C*. These genes might be considered as new targets for cancers therapies since they showed overexpression in several cancers and correlate with worse OS prognosis.

## 1. Introduction

Most proteins post-translational modifications are essential for proper cellular localization, substrate activity, and associations with other proteins. One evolutionarily conserved modification is ubiquitination, which can involve one or multiple defined lysine (K) residues or the N-terminal methionine residue (M1) [1,2,3].

Ubiquitin, a highly conserved regulatory protein (76 amino acids), plays an essential role in modulating protein functions. Ubiquitination is a post-translational modification mediated by a multi-step process which involves three different enzymes, including E1 ubiquitin-activating enzymes, E2 ubiquitin-conjugating enzymes, and E3 ubiquitin-ligating enzymes. This function leads to the proteasomal elimination of its substrate or alteration of the substrate activity, localization, and associations with other partners in its protein networks [2,4,5].

In the ubiquitin-proteasome pathway, anaphase promoting complex/cyclosome (APC/C) and the ubiquitin conjugating enzyme, E2C (UBE2C), are involved in the initiation of ubiquitin chain formation on APC/C substrates. UBE2C principally create Lys-11 (K11)-linked polyubiquitination on these substrates and then APC/C and another E2 enzyme, UBE2S, elongates and branches the ubiquitin, making more efficient proteolytic degradation signals (i.e., on mitotic cyclins) for the proteasome receptor, S5A, regulating mitosis progression. Various cellular processes are regulated by the ubiquitin system; therefore, it is expected its dysregulation results in human diseases, including cancers [6,7]. *UBE2C* overexpression has been found in different human cancers, including hepatocellular carcinoma [8], thyroid [9], colon [10], breast [11], lung [12], brain [13], and cervical cancer [14]. 

It has been shown that *UBE2C* overexpression leads to chromosomes mis-segregation and alters the cell cycle process, facilitating cell proliferation [15,16]. Moreover, it has also been reported that *UBE2C* overexpression correlates with tumor progression and poor prognosis in many tumors [9,16,17,18,19]. In this study, the expression levels of *UBE2C* were evaluated in 27 different cancers using data from the Cancer Genome Atlas (TCGA) and the Genotype-Tissue Expression (GTEx) databases. We provide evidences that *UBE2C* acts as a proto-oncogene and can be considered as a therapeutic target for most cancers. Our results indicate that *UBE2C* is overexpressed in 27 studied cancers and its overexpression correlates worsen the overall survival (OS), suggesting its involvement in tumor progression and invasion. Our study also identified a number of genes that are in the UBE2C regulatory network.

## 2. Results

### 2.1. UBE2C Overexpression in Tumors, Their Pathological Stages, and Subtypes

**Figure 1:**
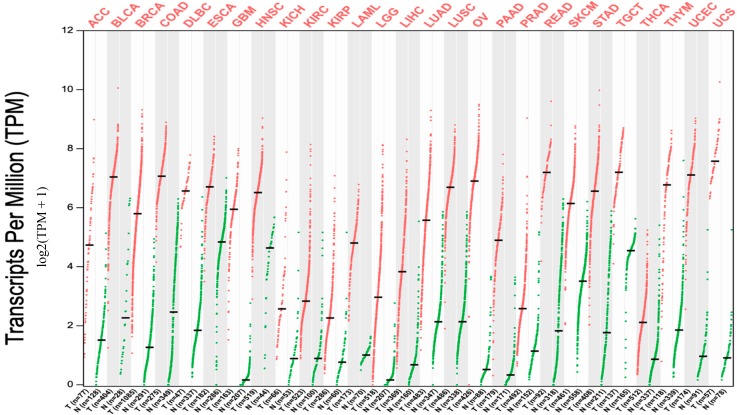
*UBE2C* expression in cancers. Expression level of *UBE2C* across 27 TCGA tumors compared to TCGA normal and GTEx data using GEPIA (Gene Expression Profiling Interactive Analysis) webserver. It is clear that in all 27 cancers there is notable upregulation of this gene. For each TCGA tumor (red), its matched normal and GTEx data (green) are given; T: tumor; N: normal; n: number. Y axis: transcript per million (log2(TPM + 1)). X axis: number of tumor and normal samples. ACC: adrenocortical carcinoma; BLCA: bladder urothelial carcinoma; BRCA: breast invasive carcinoma; COAD: colon adenocarcinoma; DLBC: lymphoid neoplasm diffuse large B-cell lymphoma; ESCA: esophageal carcinoma; GBM: glioblastoma multiforme; HNSC: head and neck squamous cell carcinoma; KICH: kidney chromophobe; KIRC: kidney renal clear cell carcinoma; KIRP: kidney renal papillary cell carcinoma; LAML: acute myeloid leukemia; LGG: brain lower grade glioma; LIHC: liver hepatocellular carcinoma; LUAD: lung adenocarcinoma; LUSC: lung squamous cell carcinoma; OV: ovarian serous cystadenocarcinoma; PAAD: pancreatic adenocarcinoma; PRAD: prostate adenocarcinoma; READ: rectum adenocarcinoma; SKCM: skin cutaneous melanoma; STAD: stomach adenocarcinoma; TGCT: testicular germ cell tumors; THCA: thyroid carcinoma; THYM: thymoma; UCEC: uterine corpus endometrial carcinoma; UCS: uterine carcinosarcoma.

Data extracted from TCGA database revealed that *UBE2C* expression was notably higher in all 27 tumor types compared to matched TCGA normal tissues and GTEx data (Figure 1). We next assessed the expression of UBE2C in normal tissue using RNA-sequencing data available from GTEx data. In particular, we compared expression levels of *UBE2C* between tumors with respect to normal matches, and data of GTEx. We found that *UBE2C* showed increased levels in all these cancers with respect to its expression in the normal tissues. The significant differences between all tumors and normal samples as a boxplot are given individually in Supplementary data Figure S1.

We next assessed the expression levels of *UBE2C* with respect to the molecular and histological subtypes of tumors, tumor grades, and other patient conditions when data are available using UALCAN. 

In urologic cancers, we found that histological subtypes of BLCA show increased expression in both papillary and non-papillary tumors compared to normal (Table 1 and Figure S2 panel 1A). In relation to its molecular subtype, all reveal upregulated compared to normal with more statistically significant values for luminal papillary, followed by basal squamous (Table 1 and Figure S2 panel 1B). In renal cancers, KIRC shows increased expression levels of *UBE2C* in all grades compared to normal, with more significant for grade 2, followed by grade 3 and 4 (Table 1 and Figure S2 panel 1C). For its subtypes, both clear cell type A (ccA) and B (ccB) (*p*-value < 10^−8^) subtypes show higher expression compared to normal, with slightly more significant for ccB (Table 1 and Figure S2 panel 1D). In KIRP tumors, all histological tumor subtypes showed *UBE2C* overexpression with high significance for type1 papillary renal cell carcinoma (RCC), followed by type2 papillary RCC (Table 1 and Figure S2 panel 1E). In PRAD tumors, the increase was statistically more significant for Gleason score 8, followed by Gleason score 9 and Gleason score 7 (Table 1 and Figure S2 panel 1F). The more statistically significant molecular signature was observed for erythroblast transformation-specific (ETS) transcription factor ERG (*ERG*) fusion, speckle type BTB/POZ protein (*SPOP*) mutation, and ETS translocation variant 1 (*ETV1*) fusion (Table 1 and Figure S2 panel 1G). In relation to the expression of *UBE2C* in metastatic PRAD based on androgen receptor (*AR*) amplification and *ERG* fusion, there is no significance difference compared to condition without these changes (Table 1 and Figure S2 panel 1H).

Compared to normal tissues in BRCA tumors, the expression of *UBE2C* was higher in all different subtypes, including triple negative breast cancer (TNBC), *HER2*-amplification, and luminal subtype (Table 1 and Figure S2 panel 2A). For its TNBC types, the statistically significant changes were seen in TNBC-mesenchymal (M), followed by TNBC-immunomodulatory (IM), TNBC-basal-like2 (BL2,), and TNBC-basal-like1 (BL1; Table 1 and Figure S2 panel 2B). The *UBE2C* expression in this BRCA was increased in all pre-, peri-, and post-menopause conditions compared to the normal tissue, but not significant compared to each other (Table 1 and Figure S2 panel 2C). In addition, *UBE2C* expression in BRCA showed high levels in all histological subtypes, with the most significant increase in infiltrating lobular carcinoma (ILC) and infiltrating ductal carcinoma (IDC; Table 1 and Figure S2 panel 2D). The expression of amplified MYC proto-oncogene (*MYC*), cyclin D1 (*CCND1*), and Erb-B2 receptor tyrosine kinase 2 (*ERBB2*) in metastatic breast cancer compared to conditions without amplification indicated no significant correlation with *UBE2C* expression (Table 1 and Figure S2 panel 2E).

In relation to digestive system tumors, COAD tumors showed increased *UBE2C* levels in adenocarcinoma and mucinous-adenocarcinoma (Table 1 and Figure S2 panel 3A). In ESCA, *UBE2C* expression was increased in both subtypes, including adenocarcinoma and squamous-cell-carcinoma (Table 1 and Figure S2 panel 3B). The expression in HNSC in all grades is higher than in normal tissue, particularly for grade 3 tumors (Table 1 and Figure S2 panel 3C). Also, the expression based on human papilloma virus (HPV) status showed a more statistically significant increase in HPV negatives than positives compared to normal in this cancer (Table 1 and Figure S2 panel 3D). All grades of LIHC tumors showed high *UBE2C*, with grade 2 and 3 more statically significant (Table 1 and Figure S2 panel 3E). In PAAD tumors based on patients’ drinking habits, the expression was more statistically significant only for occasional drinkers and weekly drinkers (Table 1 and Figure S2 panel 3F). On the basis of diabetes status, the expression in non-diabetics was more significant than diabetics when compared to normal (Table 1 and Figure S2 panel 3G), but their comparison with each other was not statistically significant. On the basis of pancreatitis status, the expression was more significant in non-pancreatitis than pancreatitis compared to their matched normal (Table 1 and Figure S2 panel 3H), but the expression was not significant when these two were compared to each other. In READ, both adenocarcinoma and mucinous-adenocarcinoma showed a significant increase of expression, with the first one more statistically significant (Table 1 and Figure S2 panel 3I). STAD tumors showed high *UBE2C* expression in all conditions with high significance in tumors without *H. pylori* infection (Table 1 and Figure S2 panel 3J), but the comparison with each other was not statistically significant. For its histological subtypes, all of them showed increased levels, with more statistically significant for intestinal adenocarcinoma-not otherwise specified (NOS), intestinal adenocarcinoma tubular, and adenocarcinoma NOS with the same change, followed by adenocarcinoma diffuse and intestinal adenocarcinoma mucinous (Table 1 and Figure S2 panel 3K). Also, all grades of STAD show significant *UBE2C* overexpression compared to normal (Table 1). 

Regarding lung cancers, while approximately all histological subtypes of LUAD tumors showed increased *UBE2C* expression, the increased level was more statistically significant for lung adenocarcinoma mixed type, followed by lung adenocarcinoma NOS (Table 1 and Figure S2 panel 4A). Regarding LUSC, for its histological subtypes, the increase was more significant for LUSC NOS, followed by lung basaloid squamous cell carcinoma (Table 1 and Figure S2 panel 4B).

All histological subtypes of UCEC tumors showed a notable *UBE2C* increase, which was more statistically significant for serous and endometrioid and then mixed serous and endometrioid (Table 1 and Figure S2 panel 5A). For this cancer type, the *UBE2C* expression was more significant for post-menopause (Table 1 and Figure S2 panel 5B).

When we inspected the contribution of smoking habits of cancer patients to *UBE2C* expression, we found no difference between smokers and non-smokers in BLCA patients. Nevertheless, the *UBE2C* expression level was higher in reformed smokers (>15 years) compared to non-smokers (Figure S2 panel 6A). Regarding smoking habits in ESCA, the expression levels in all conditions were higher than normal, but was more significant in smokers than non-smokers (Figure S2 panel 6B). In LUAD, the expression in smokers (reformed smoker2, smokers, and reformed smoker1) showed more significant values than non-smokers (Figure S2 panel 6C). Regarding LUSC patients based on smoking habits, the expression in all categories showed an increase compared to normal, which was more significant for reformed smoker1, smoker, and reformed smoker2. However, there was no statistically significant difference between smokers and non-smokers in LUSC cancer (Figure S2 panel 6D). 

**Figure 2:**
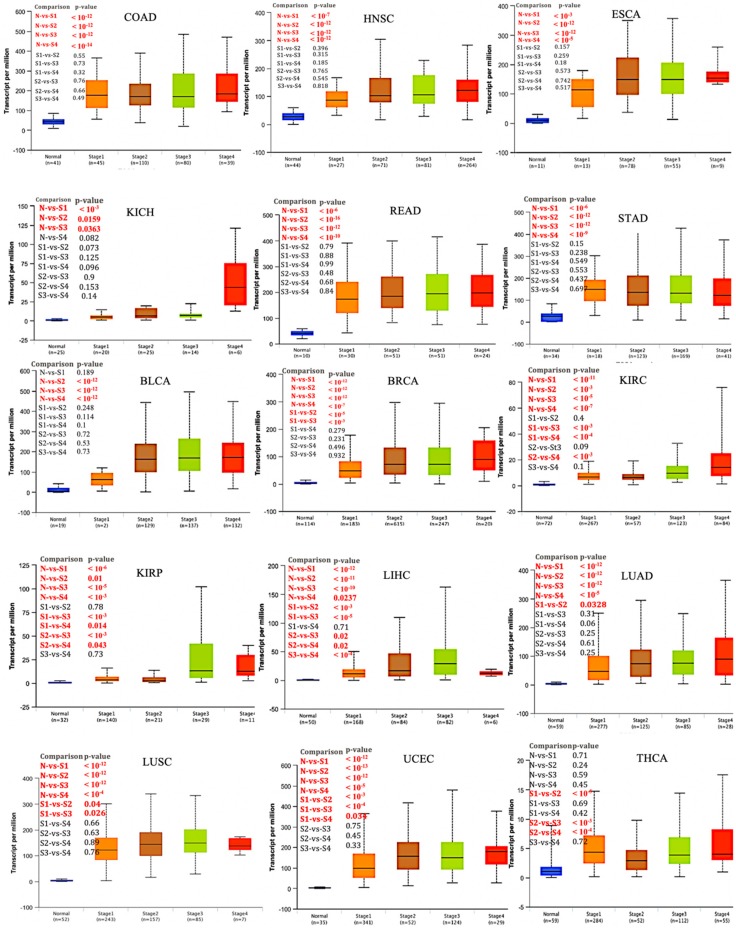
*UBE2C* expression based on individual pathological cancer stage. Box plot reveals that the over-expression of *UBE2C* may have role in initiation of COAD, HNSC, ESCA, KICH, READ, STAD, and BLCA, but not in progression since significant changes were observed only between normal and pathological stages not between each stage. The expression of *UBE2C* in BRCA, KIRC, KIRP, LIHC, LUAD, LUSC, and UCEC shows its involvement in both cancer initiation and progression. Regarding THCA, while there were no significant changes between normal and pathological stages, it seems that *UBE2C* is involved in progression from stage 1 to 2 and then 2 to 3. Y axis: transcript per million, X axis: pathological cancer stages with the number of samples in each stage in parenthesis. N: normal, S: stage.

We next investigated *UBE2C* expression on the basis of patients’ pathological stage in TCGA cancer types. We found that in COAD, ESCA, HNSC, KICH, READ, STAD, and BLCA, *UBE2C* expression levels were significantly higher in early-stages (Figure 2, *p*-value < 0.05). This indicates a possible involvement of *UBE2C* in the initiation of cancer. Furthermore, the expression in BRCA, KIRC, KIRP, LIHC, LUAD, LUSC, and UCEC was higher in late-stage cancers compared to early stages, representing a possible role of *UBE2C* in cancer progression and invasion (Figure 2, cancer without and/or small numbers of normal matches (when there is only one sample in each stage) were excluded from this analysis). 

### 2.2. Role of UBE2C Overexpression in Cancer Prognosis

**Figure 3:**
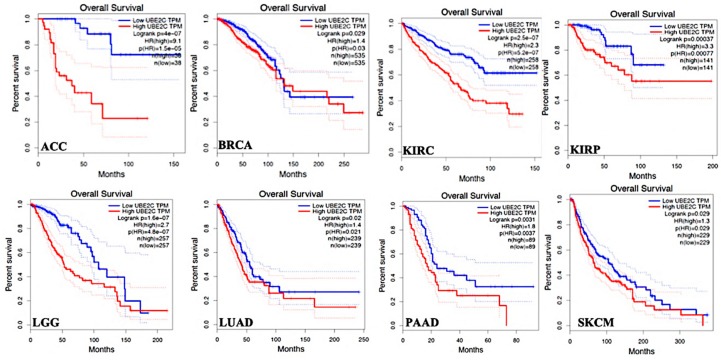
OS time between *UBE2C* higher-expression-level and *UBE2C* lower-expression-level tumors in TCGA tumor types with shorter overall survival time and worse OS prognosis. Red line shows the cases with highly expressed *UBE2C* and blue line is indicated for the cases with lowly expressed *UBE2C*. HR: hazard ratio.

The OS time between *UBE2C* higher-expression-level and *UBE2C* lower-expression-level tumors were compared in TCGA tumor types and data revealed a shorter OS with worse prognosis in patients with *UBE2C* higher expression levels compared to its lower expression levels in the following cancers: ACC, BRCA, KIRC, KIRP, LGG, LUAD, PAAD, and SKCM (Figure 3, only cancers with significant changes, *p*-value < 0.05, a shorter OS with worse prognosis, are given).

**Figure 4:**
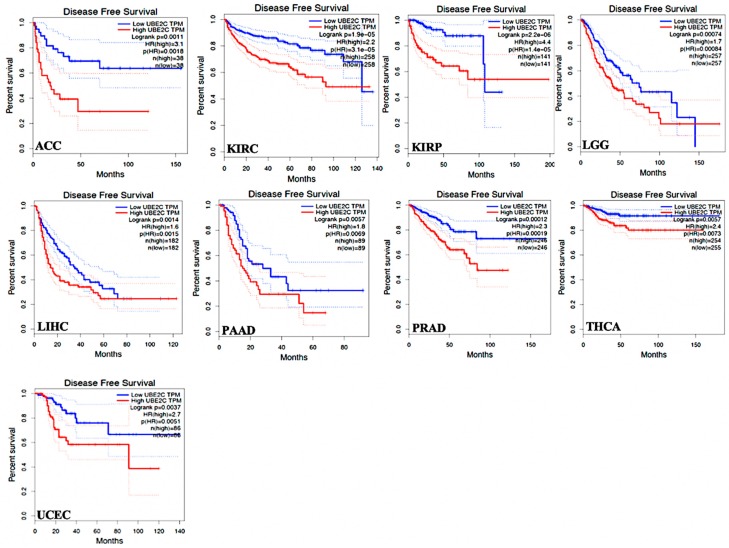
DFS time between *UBE2C* higher-expression-level and *UBE2C* lower-expression-level tumors in the TCGA tumor types with worse prognosis. Red line shows the cases with highly expressed *UBE2C* and blue line is indicated for the cases with lowly expressed *UBE2C*. HR: hazard ratio.

Regarding DFS time in the TCGA tumor types, data showed that *UBE2C* higher-expression levels led to worse DFS prognosis in comparison to its lower expression in the following tumors: ACC, KIRC, KIRP, LGG, LIHC, PAAD, PRAD, THCA, and UCEC. (Figure 4, only cancers with significant changes, *p*-value < 0.05, worse DFS prognosis, are given). These data demonstrate that *UBE2C* overexpression results in poor clinical outcomes in the above tumors.

### 2.3. Gene Expression Correlation between UBE2C and Other Genes in Cancers

Our study revealed that the expression of *UBE2C* has a moderate to very strong positive correlation with other genes in 27 cancers (Supplementary Table S1 file, all information related to coefficient correlation and *p*-value are given in this file and different colors are used to distinguish the correlation as follows: Strong and very strong positive correlation in green; medium positive correlation in black, weak and very weak positive correlation in red; and negative correlation in violet). As seen in Supplementary Table S1 file and Table 2, the positive *UBE2C* expression correlations are strong to very strong (R between 0.6 and 1 and *p*-value < 0.05) for the following genes in all 27 cancers: MYB proto-oncogene like 2 (*MYBL2*), trophinin associated protein (*TROAP*), cell division cycle 20 (*CDC20*), centromere protein A (*CENPA*), kinesin family member C1 (*KIFC1*), cyclin dependent kinase 1 (*CDK1*), kinesin family member 4A (*KIF4A*), and kinesin family member 20A (*KIF20A)*. In addition, the following genes showed a strong to very strong positive expression in correlations with *UBE2C* in 26 cancers, but moderate positive correlations (R between 0.4 and 0.59 and *p*-value < 0.05, Table 2 and Table S1 file) in one cancer, including TPX2, microtubule nucleation factor (*TPX2)*, polo like kinase 1 (*PLK1)*, aurora kinase B (*AURKB*), non-SMC condensin I complex subunit G (*NCAPG*), cyclin B1 (*CCNB1*), spindle and kinetochore associated complex subunit 3 (*SKA3)*, and kinesin family member 18B (*KIF18B)*. Moreover, some genes showed strong to very strong positive expression correlations with *UBE2C* in several cancers while moderate correlations were found in a few cancers among 27 studied cancers as indicated in the Table S1 file and Table 2. Among the 27 cancers, most negative correlations between *UBE2C* expression and different genes were observed in the TGCT cancer mentioned in Table S1 file (in violet) and Table 2, with a strong negative correlation for Testis-specific Y-encoded-like protein 2 (*TSPYL2*), ATR serine/threonine kinase (*ATR*)*,* and CYLD lysine 63 deubiquitinase (*CYLD*).

Regarding genes which have protein products that have transcription factor binding sites on both the promoter and enhancer regions of *UBE2C,* we found forkhead box M1 (*FOXM1*, two sites with transcription start site (TSS) distance of +0.1 kb and +158.3 kb), E2F transcription factor 1 (*E2F1*, TSS distance: +158.3 kb), RAD51 recombinase (*RAD51*, two sites with TSS distance of +551.6 kb and +123.8 kb), and BRCA1 DNA repair associated (*BRCA1*, TSS distance: +158.3 kb). It is worthwhile to note that only *FOXM1, E2F1,* and *RAD51* showed positive correlations with *UBE2C* in all 27 cancers. These three genes showed similar strong to very strong positive correlations with *UBE2C* in the different cancers given in Table 2 and Supplementary data Table S1.

In relation to the expression correlation between *UBE2C* and tumor suppresser genes including BUB1 mitotic checkpoint serine/threonine kinase B (*BUB1B*)*, BRCA1,* BRCA2 DNA repair associated (*BRCA2*), checkpoint kinase 2 (*CHK2*), ATM serine/threonine kinase (*ATM*), *ATR,* tumor protein p53 (*TP53*), *CYLD*, and *TSPYL2,* the positive correlation between *BUB1B* and *UBE2C* was seen in 26 cancers, but not in TGCT cancer (Table 2 and Supplementary Data Table S1). Regarding *BRCA1* and *BRCA2,* both of these genes showed similar strong positive correlations with *UBE2C* in different cancers (Table 2 and Table S1 file), but a negative correlation was observed for BRCA1 in THCA and for BRCA2 in TGCT (Table 2 and Supplementary Data Table S1). *CHK2* and *TP53* genes showed a positive correlation with *UBE2C* in several cancers (Table 2 and Table S1 file) while *CHK2* showed a strong negative correlation in KICH and *TP53* revealed a very weak negative correlation in this cancer. Three tumor suppresser genes, including *CYLD*, *ATM*, and *TSPYL2*, showed a negative correlation with *UBE2C* in several cancers with a similar moderate to strong negative correlation for them in the following cancers: LUSC, READ, UCEC, OV, and UCS. It is worthy to note that *TSPYL2* showed down-regulation in most of the 27 cancers (Supplementary Data Figure S3) and also very weak to strong negative correlation with *UBE2C* in most of these studied cancers (Table S1 file and Table 2). Among the genes with a positive expression correlation with *UBE2C,* some genes that can be considered as target genes in several cancers. For example, mitochondrial fission regulator 2 (*MTFR2* also called *FAM54A*, only limited studies in GBM [20] and ovarian cancer [21]), meiotic nuclear divisions 1 (*MND1*, limited studies in breast [22] and ovarian cancers [23]), family with sequence similarity 72 member D (*FAM72D* only one study in GBM [24]), and POC1 centriolar protein A (*POC1A*, limited reports in bladder [25], brain [26], and breast cancer [27]). As seen in Table 3 and Supplementary Figure S4 file, they showed an overexpression, with alterations across different pathological cancer stages and a worse OS prognosis in several cancers. 

### 2.4. UBE2C Protein Network 

**Figure 5:**
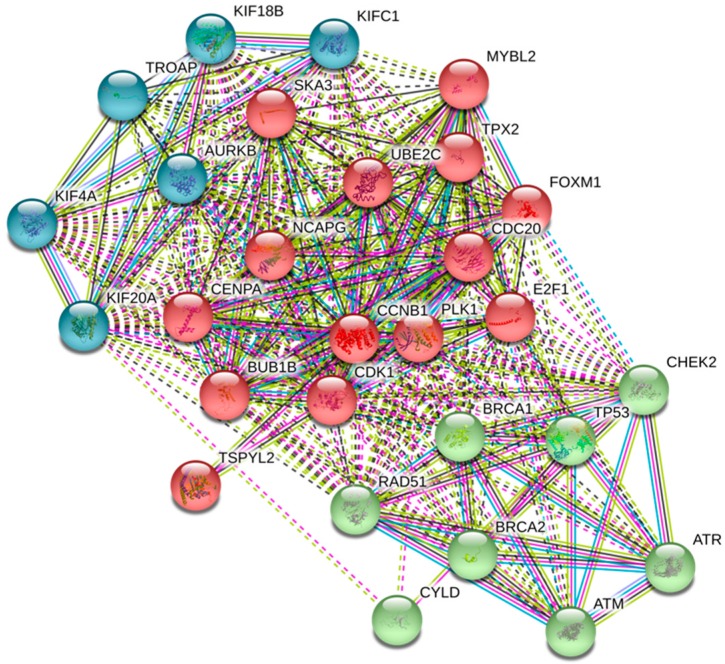
UBE2C protein network. Proteins with a strong to very strong positive correlation with UBE2C in most of the 27 cancers and some tumor suppressors are clustered in three categories based on the kmeans clustering option in STRING. In the cluster indicated in blue, different members of kinesin Family (KIF20A, KIF18B, KIFC1, and KIF4A) are included, which function as microtubule-dependent molecular motor, AURKB (a serine/threonine-protein kinase component of the chromosomal passenger complex, CPC) with an essential role in the regulation of mitosis, and TROAP involved in cell adhesion molecule complex. Another cluster shown in red (TPX2, UBE2C, PLK1, CDK1, CENPA, CDC20, MYBL2, BUB1B, CCNB1, NCAPG, SKA3, E2F1, FOXM1, and TSPYL2) is involved in different vital cellular processes, such as cell cycle, regulation of cellular metabolic process, cellular protein modification, signaling, chromosome organization, histone modification, and others as described in Table S2 file. The last cluster in green includes major tumor suppressor genes, including BRCA2, BRCA1, CHEK2, ATR, ATM, TP53, RAD51, and CYLD.

Genes with strong or very strong positive correlations with *UBE2C* expression in the 27 cancers (see Methods Section, Table 2, and Supplementary Table S1) and also some negative correlations with important tumor suppressor genes were identified in the UBE2C protein network. Data from STRING database revealed that all these proteins (products of all genes listed in Table S1 file) are in the same protein network (Figure 5, only proteins with strong and very strong positive correlations with *UBE2C* in most of the 27 cancers, some tumor suppressor proteins, and protein with TF bindings site on *UBE2C* are shown). Proteins in this network are involved in different pathways, including cell cycle, oocyte meiosis, p53 signaling pathway, double-strand break repair, oocyte development and differentiation, FoxO signaling pathway, ubiquitin mediated proteolysis, cellular senescence, and progesterone-mediated oocyte maturation among others (Table 4 and Supplementary data Table S2). All pathways that UBE2C is mainly involved with are indicated in red (Supplementary Table S2). Proteins with TF binding sites on both the promoter and enhancer regions of *UBE2C* (FOXM1, E2F1, RAD51, and BRCA1) are also involved in the UBE2C network. As described in Table 4 and in Supplementary data Table S2, these proteins were found to also be involved in most pathways related to the UBE2C protein network. Among the UBE2C protein partners, TSPYL2 (a member of the testis-specific protein Y-encoded) is a tumor suppressor protein which acts in the chromatin remodeling process. This protein is also involved in most pathways with the involvement of UBE2C (Supplementary data Table S2). As *TSPYL2* was under-expressed in most cancers (Supplementary data Figure S3) and showed negative correlations with *UBE2C* that were very weak to moderate (Table 2 and Supplementary data Table S1), we analyzed the RNA–RNA association between TSPYL and UBE2C using RNAup webserver and also RNA–protein interactions using RPISeq. These analyses showed that TSPYL2 is not only involved in the UBE2C protein network, but also in RNA–RNA interactions and RNA–protein interactions with UBE2C (Figure S5 panel A and B). Moreover, these two proteins were in the same subcellular localization. We also found a D-box (one of the recognition amino acid sequences identified by APC/C in the ubiquitin–proteasome pathway) in amino acids 45 to 48 in TSPYL2 protein using the GPS-ARM tool (Figure S5 panel C).

## 3. Discussion

UBE2C is a member of the E2 ubiquitin-conjugating enzyme family, it plays a key role in the ubiquitination system in cooperation with APC/C. It is involved in mitotic cyclin B degradation, promoting the transition from the M phase to the G1 phase of the cell cycle [28,29]. Therefore, it is likely that aberrant *UBE2C* overexpression, leading to changes in ubiquitination, might be involved in uncontrolled cell proliferation, which is one of the main features of cancers.

The current study shows, for the first time, a global analysis of *UBE2C* expression in a wide array of tumors. Our results demonstrated that *UBE2C* is upregulated in all 27 different cancers examined and this is in agreement with previous reports showing the increased somatic expression of *UBE2C* in various tumor types. Indeed, the overexpression of *UBE2C* was previously reported in hepatocellular carcinoma, thyroid, colon, breast, and lung cancer [8,9,10,11,12,13] and our data confirmed this upregulation (Figure 1). To give an example, in a study conducted by Qin et al., the overexpression of *UBE2C* was found in breast cancer, including the basal-like (BL) subtype [30], and our study also showed this significant upregulation in both BL1 and BL2. Importantly, our investigation showed that overexpression of *UBE2C* is a common feature of all 27 human cancers tested in this study, suggesting it acts as a proto-oncogene. Moreover, the current study revealed the significant *UBE2C* overexpression across the histological and molecular subtypes of different tumors mentioned in the results and also possible associations between this expression and different patient conditions, such as drinking and smoking habits in PAAD and LUAD, respectively. *UBE2C* expression, based on patients’ pathological stages, showed that *UBE2C* overexpression can be involved in tumor progression and invasion. Our data broaden the observations of previous reports [15,30,31].

We showed that patients with higher expression of *UBE2C* had a shorter overall survival time and worse prognosis, and *UBE2C* higher-expression levels also resulted in worse DFS prognosis in many cancers, confirming that *UBE2C* overexpression results in poor clinical outcomes in many tumors. As an example, Qin et al. found that the *UBE2C* upregulation was associated with poor prognosis in breast cancer [30], and our data also revealed this issue as shown in Figure 3.

Furthermore, our study identified coexpression genes associated with the UBE2C protein network. Those genes with strong and very strong positive correlations with *UBE2C* expression in all cancers (Table 2) are involved in the cell cycle process. These include members of the kinesin family (*KIF20A*, *KIF18B*, *KIFC1*, and *KIF4A*), with roles in mitotic spindle maintenance, chromosome segregation, and microtubule depolarization [32,33]; *AURKB* as a regulator of chromosome segregation during mitosis [34]; *TROAP* (a member of the cell adhesion molecule complex) with a role in centrosome integrity during cell cycle progression [35]; *TPX2* (a microtubule-associated protein) having a key function in mitotic spindle formation [36]; *PLK1* as a regulator of cell division and maintenance of genome stability and spindle assembly, and DNA damage response) [37,38]; *CDK1* (a cyclin-dependent kinase), involved in cell division with important functions in mitosis and driving cells into the S phase [39]; *CENPA* (a key component of the inner kinetochore plate), playing a role in chromosome segregation during oocyte meiosis) [40]; *CDC20* as an activator of APC/C during mitosis for mitosis progression [41]; *MYBL2*, involved in cell cycle progression, cell survival, and cell differentiation [42]; *BUB1B* as a vital component of the mitotic checkpoint complex [43]; *CCNB1*, having an essential role in the transition of the cell cycle from G2 phase to mitosis [44,45]; *NCAPG* (a mitosis-related chromosome condensation protein) involved in the condensin I complex [46]; *SKA3*, controlling the correct exit from meiosis, migration of meiotic spindle, and stability of anaphase spindle [47]. Our data highlight the UBE2C network as one of the major protein networks involved in cancer and further investigation on their function in tumors might shed light onto new therapeutic strategies for cancer. 

Interestingly, we also highlighted as *UBE2C* coxpressed genes the transcription factors, FOXM1, E2F1, and RAD51, which have their binding sites on the *UBE2C* promoter and enhancer regions, suggesting they are transcriptional regulators of this gene. Our results are in agreement with a previous report of a positive association of FOXM1 with *UBE2C* in normal tissues and in tumors [48]. The identification of RAD51, which has a key role in the homologous recombination and repair of DNA [49], and mainly E2F1 could be particularly relevant since E2F1 contributes to the activation of genes involved in G1/S progression [50,51,52,53]. 

We also found a significant correlation between *UBE2C* and other important regulators of the cell cycle, including *CDC20*, which was previously identified as being co-expressed with *UBE2C* [54]. These data suggest that other genes identified in this study as part of the UBE2C protein network might play a role in many tumors. In particular, our data highlighted *MTFR2, MND1, FAM72D,* and *POC1A* as genes whose expression correlates with worse OS prognosis, suggesting their possible involvement in tumor progression and invasion. 

In conclusion, the current study showed that *UBE2C* can be considered as a general tumor marker and study of its related pathways can help to discover common therapeutic targets for cancers. However, further functional studies are required to clarify the role of *UBE2C* in cancers.

## 4. Materials and Methods

In the current study, our investigations were performed using different bioinformatics tools and databases, including GEPIA [55] (a webserver which extracts data from the Cancer Genome Atlas (TCGA) data portal and the GTEx database of normal tissues. http://gepia.cancer-pku.cn), UALCAN (an interactive web portal for the in-depth analysis of TCGA gene expression data, http://ualcan.path.uab.edu) [56], and STRING databases (functional protein association networks (https://string-db.org/) [57]).

Here, we report the following investigations: *UBE2C* expression levels across all cancers and their subtypes, its differential gene expression analysis at different pathological stages, correlation between its expression and cancer prognosis in cancers, overall survival (OS) and the disease-free survival (DFS) analysis on the basis of *UBE2C* gene expression, investigation of genes with similar *UBE2C* expression patterns, and their associations in the UBE2C protein network.

To investigate *UBE2C* expression across 27 human tumor types compared to normal matches, we used the GEPIA webserver. One advantage of GEPIA is that it also uses normal data from the GTEx project to provides a reliable baseline for comparison. In most cancer research, normal tissues are prepared from areas adjacent to tumors, but they may be pre-cancerous tissue and not truly normal, healthy tissue. *UBE2C* expression between tumors, their matched normal, and data from the GTEX database in 27 tumor types were compared. These tumors included ACC, BLCA, BRCA, COAD, DLBC, ESCA, GBM, HNSC, KICH, KIRC, KIRP, LAML, LGG, LIHC, LUAD, LUSC, OV, PAAD, PRAD, READ, SKCM, STAD, TGCT, THCA, THYM, UCEC, and UCS. Six tumor types were excluded from the study (where differential expression was considered) due to their small numbers (13 and lower) or lack of normal samples, including cholangio carcinoma (CHOL), cervical squamous cell carcinoma and endocervical adenocarcinoma (CESC), mesothelioma (MESO), pheochromocytoma and paraganglioma (PCPG), sarcoma (SARC), and uveal melanoma (UVM). Regarding parameter options, we used the ANOVA statistical method for differential gene expression analysis, selected log2(TPM + 1) transformed expression data for plotting, TCGA tumors compared to TCGA normal and GTEx normal for matched normal data in plotting, |log2FC| cutoff of 1, and a q-value cutoff of 0.01. Also, for cancers with different subtypes and conditions, we analyzed them using the UALCAN webserver.

To provide *UBE2C* expression box plots on the basis of patients’ pathological stage (stage I, stage II, stage III, and stage IV group) in TCGA cancer types, we used the UALCAN webserver to get data from TCGA. In this analysis, cancers without normal matches or below two numbers in each stage were excluded from analysis.

Overall survival (OS) and the disease-free survival (DFS) analysis were also performed on the basis of *UBE2C* gene expression. Regarding hypothesis test, the GEPIA considers the Log-rank test. For this, we selected a hazards ratio (HR) based on the Cox PH model and also the 95% confidence interval information to show the 95% confidence interval (CI) as the dotted line. The *UBE2C* expression threshold of 50% (median value) was determined to split the *UBE2C* high-expression and low-expression cohorts. Therefore, samples with *UBE2C* expression levels higher and lower than 50% were applied as the high-expression cohort (cutoff-high) and the low-expression cohort (cutoff-low), respectively.

Correlation analysis between *UBE2C* and other genes was performed by pair-wise gene expression correlation analysis with the expression data of TCGA and GTEx, using the method of the Pearson correlation coefficient. At first, we searched for moderate, strong, and very strong *UBE2C* expression correlations (the Pearson correlation coefficient between 0.4 and 1) with other genes on average in all 27 caners. Then, we investigated the *UBE2C* expression correlations with each gene individually in each cancer to see the exact correlation. We considered the following correlation coefficients: 0.00–0.19 as very weak, 0.20–0.39 as weak, 0.40–0.59 as fairly strong (also called moderate), 0.60–0.79 as strong, and 0.80–1.0 as very strong. 

To provide the UBE2C protein network, the STRING database was used and most genes with medium to very strong correlations with *UBE2C* (extracted from TCGA cancer types using GEPIA) and also some important tumor suppresser proteins, such as ATR, ATM, BUB1B, BRCA1/2, CHK2, and CYLD, were searched in STRING.

To predict the RNA–RNA association and RNA–protein interaction between TSPYL and UBE2C, we used the RNAup webserver (http://rna.tbi.univie.ac.at//cgi-bin/RNAWebSuite/RNAup.cgi) and RPISeq (http://pridb.gdcb.iastate.edu/RPISeq/), respectively. Also, to search for a D-box (one of the recognition amino acid sequences to be identified by APC/C in the ubiquitin–proteasome pathway) in TSPYL2 protein, we used the GPS-ARM tool (http://arm.biocuckoo.org/down.php). 

## Figures and Tables

**Table 1:** Statistically significant *UBE2C* overexpression based on histological, molecular subtypes, and different patient statuses (only findings with *p*-value < 0.05 are given).

Tumor	Histological Subtypes	Molecular Subtypes	Tumor Grade	Other Patient Conditions
**BLCA**	N-vs.-Papillary tumors: *p* < 10^−12^ N-vs.-NonPapillary tumors: *p* < 10^−12^	N-vs.-Neuronal: *p* < 10^−8^; N-vs.-Basal squamous: *p* < 10^−12^; N-vs.-Luminal: *p* < 10^−10^; N-vs.-Luminal_Infiltrated: *p* < 10^−9^; N-vs.-Luminal_Papillary: *p* < 10^−12^		
**KIRC**		N-vs.-ccA subtype: *p* = 10^−8^ N-vs.-ccB subtype: *p* < 10^−8^	N-vs.-G 1: *p* < 10^−3^ N-vs.-G 2: *p* < 10^−11^ N-vs.-G 3: *p* < 10^−9^ N-vs.-G 4: *p* < 10^−6^	
**KIRP**	N-VS.-Type1 PRCC: *p* < 10^−12^ N-VS.-Type2 PRCC: *p* < 10^−7^ N-VS.-KIRP CIMP: *p* < 10^−3^ N-VS.-Unclassified PRCC: *p* < 10^−3^			
**PRAD**		N-vs.-ERG fusion: *p* < 10^−5^ N-vs.-ETV1 fusion: *p* < 10^−3^ N-vs.-SPOP mutation: *p* < 10^−5^		N-vs.-Gleason score 7: *p* < 10^−3^ N-vs.-Gleason score 8: *p* < 10^−5^ N-vs.-Gleason score 9: *p* < 10^−4^
**BRCA**	N-vs.-IDC: *p* < 10^−12^ N-vs.-ILC: *p* < 10^−12^ N-vs.-Mixed: *p* < 10^−6^ N-vs.-Other: *p* < 10^−7^ N-vs.-Mucinous: *p* < 10^−3^ N-vs.-Metaplastic: *p* < 10^−3^ N-vs.-Medullary: *p* < 10^−3^	N-vs.-Luminal: *p* < 10^−12^ N-vs.-HER2 Positive: *p* < 10^−10^ N-vs.-TNBC: p < 10^−12^ N-vs.-TNBC-BL1: *p* < 10^−7^ N-vs.-TNBC-BL2: *p* < 10^−8^ N-vs.-TNBC-IM: *p* < 10^−8^ N-vs.-TNBC-LAR: *p* < 10^−3^ N-vs.-TNBC-MSL: *p* < 10^−4^ N-vs.-TNBC-M: *p* < 10^−10^ N-vs.-TNBC-UNS: *p* < 10^−5^		N-vs.-Pre-Menopause: *p* < 10^−12^ N-vs.-Peri-Menopause: *p* < 10^−6^ N-vs.-Post-Menopause: *p* < 10^−12^
**COAD**	N-vs.-Adenocarcinoma: *p* < 10^−12^ N-vs.-Mucinous-adenocarcinoma: *p* < 10^−11^			
**ESCA**	N-vs.-Adenocarcinoma: *p* < 10^−12^ N-vs.-Squamous-cell-carcinoma: *p* < 10^−12^		G 2-vs.-G 3: *p* < 10^−8^	
**HNSC**			N-vs.-G 1: *p* < 10^−12^ N-vs.-G 2: *p* < 10^−12^ N-vs.-G 3: *p* < 10^−12^ N-vs.-G 4: *p* < 10^−5^	N-vs.-HPV+ve: *p* < 10^−11^ N-vs.-HPV-ve: *p* < 10^−12^
**LIHC**			N-vs.-G 1: *p* < 10^−5^ N-vs.-G 2: *p* < 10^−12^ N-vs.-G 3: *p* < 10^−12^ N-vs.-G 4: *p* < 10^−3^	
**PAAD**			G1-vs.-G 2: *p* = 0.014 G1-vs.-G3: *p* = 0.0062	N-vs.-Weekly Drinker: *p* = 0.025 N-vs.-Occasional Drinker: *p* < 10^−3^ N-vs.-Diabetic: *p* < 10^−4^ N-vs.-NonDiabetic: *p* < 10^−7^ N-vs.-Pancreatitis: *p* < 10^−4^ N-vs.-NoPancreatitis: *p* < 10^−7^
**READ**	N-vs.-Adenocarcinoma: *p* < 10^−12^ N-vs.-Mucinous-adenocarcinoma: *p* < 10^−3^			
**STAD**	N-vs.-Adenocarcinoma (NOS): *p* < 10^−12^ N-vs.-Adenocarcinoma (Diffuse): *p* < 10^−10^ N-vs.-Adenocarcinoma (Signet Ring): *p* < 10^−3^ N-vs.-Intestinal Adenocarcinoma (NOS): *p* < 10^−12^ N-vs.-IntestinalAdenocarcinoma (Tubular): *p* < 10^−12^ N-vs.-IntestinalAdenocarcinoma (Mucinous): *p* < 10^−4^ N-vs.-IntestinalAdenocarcinoma (Papillary): *p* = 0.017		N-vs.-G 1: *p* = 0.0057 N-vs.-G 2: *p* < 10^−12^ N-vs.-G 3: *p* < 10^−12^ G 1-vs.-G 3: *p* = 0	N-vs.-Tumors (with *H. pylori* infection): *p* < 10^−4^ N-vs.-Tumors (without *H. pylori* infection): *p* < 10^−12^ N-vs.-Tumors (Not available): *p* < 10^−12^
**LUAD**	N-vs.-NOS: *p* < 10^−12^ N-vs.-Mixed: *p* < 10^−14^ N-vs.-LBC-NonMucinous: *p* < 10^−4^ N-vs.-SolidPatternPredominant: *p* = 0.019 N-vs.-Acinar: *p* < 10^−5^ N-vs.-LBC-Mucinous: *p* < 10^−5^ N-vs.-Mucinous carcinoma: *p* < 10^−3^ N-vs.-Papillary: *p* < 10^−4^			
**LUSC**	N-vs.-NOS: *p* < 10^−12^ N-vs.-Basaloid: *p* < 10^−5^ N-vs.-Papillary: *p* = 0.018			
**UCEC**	N-vs.-Endometrioid: *p* < 10^−12^ N-vs.-Serous: *p* < 10^−12^ N-vs.-Mixed serous and endometrioid: *p* < 10^−7^			N-vs.-Pre-Menopause: *p* < 10^−9^ N-vs.-Peri-Menopause: *p* < 10^−4^ N-vs.-Post-Menopause: *p* < 10^−12^
**THCA**	Classical-VS.-Follicular: *p* < 10^−5^ Tall-VS.-Follicular: *p* < 10^−3^			

N: normal; *p*: *p*-value; amp: amplification; G: grade. All details are given in Supplementary Figure S2.

**Table 2:** Expression correlation between *UBE2C* and various genes.

**Genes with Strong to very Strong Positive *UBE2C* Expression Correlations in all 27 Cancers**	**Genes with Strong to very Strong Positive *UBE2C* Expression Correlations in 26 Cancers, but Moderate Positive Correlation in One Cancer**	**Genes with Strong to very Strong Positive *UBE2C* Expression Correlations in Several Cancers, but Moderate in Few Cancers**	**Gene with Negative Correlation with *UBE2C* Expression in TGCT Cancer**
*MYBL2, TROAP, CDC20, CENPA, KIFC1, CDK1, KIF4A,* and *KIF20A*.	*TPX2, PLK1, AURKB, NCAPG*, *CCNB1*, *SKA3*, and *KIF18B*.	*CDCA3, HJURP, UBE2T, MAD2L1, DLGAP5, MELK, RNASEH2A, KIF23, FAM72D,* and *FEN1*	*KIF2C, CDCA5, CDC25C, BUB1*, *MTFR2, KIF15, CDKN3, CKS2, BUB1B, ASF1B, NUF2, PLK4, TTK, POC1A, UBE2S, AUNIP, NEK2, DSN1, BRCA2*, *CYLD*, *ATM*, *ATR*, and *TSPYL2*
**Gene with negative correlation with *UBE2C* expression in different cancers**			**Positive expression correlation between UBE2C and genes having TF binding sites on both promoter and enhancer regions of *UBE2C* in different cancers**
***BRCA1*** in THCA ***BRCA2*** in TGCT ***CHK2*** in KICH, and THYM ***CYLD*** in COAD, KIRP, LUAD, LUSC, PRAD, READ, SKCM, STAD, THCA, UCEC, ACC, LAML, OV, UCS, THYM, GBM, TGCT ***ATM*** in BLCA, BRCA, COAD, KIRC, LUAD, LUSC, PAAD, READ, STAD, THCA, UCEC, ACC, OV, UCS, THYM, GBM, HNSC, TGCT ***ATR*** in LUAD, SKCM, THCA, UCEC, ACC, OV, UCS, TGCT ***TP53*** in KICH ***TSPYL2*** in BLCA, BRCA, COAD, ESCA, KICH, LIHC, LUAD, LUSC, PAAD, PRAD, READ, SKCM, STAD, THCA, UCEC, ACC, OV, UCS, THYM, LGG, GBM, HNSC, TGCT			**BRCA** (*FOXM1:* *R* = 0.69; *E2F1*: *R* = 0.7; *RAD51*: *R* = 0.67) **ESCA** (*FOXM1*: *R* = 0.79; *E2F1*: *R* = 0.82; *RAD51*: *R* = 0.83) **KICH** (*FOXM1*: *R* = 0.84; *E2F1*: *R* = 0.83; *RAD51*: *R* = 0.63) **KIRC** (*FOXM1*: *R* = 0.73; *E2F1*: R = 0.7; *RAD51*: *R* = 0.72) **LUSC** (*FOXM1*: *R* = 0.72; *E2F1*: *R* = 0.67; *RAD51*: *R* = 0.7) **PAAD** (*FOXM1*: *R* = 0.82; *E2F1*: *R* = 0.88; *RAD51*: *R* = 0.86) **READ** (*FOXM1*: *R* = 0.73; *E2F1*: *R* = 0.86; *RAD51*: *R* = 0.74) **SKCM** (*FOXM1*: *R* = 0.76; *E2F1*: *R* = 0.7, *RAD51*: *R* = 67) **UCEC** (*FOXM1*: *R* = 0.7; *E2F1*: *R* = 0.75; *RAD51*: *R* = 0.66) **ACC** (*FOXM1*: *R* = 0.84; *E2F1*: *R* = 0.69; *RAD51*: *R* = 0.86) **DLBC** (*FOXM1*: *R* = 0.89; *E2F1*: *R* = 0.82; *RAD51*: *R* = 0.9) **THYM** (*FOXM1*: *R* = 0.72; *E2F1*: *R* = 0.84; *RAD51*: *R* = 0.82) **LGG** (*FOXM1*: *R* = 0.9; *E2F1*: *R* = 0.8; *RAD51*: *R* = 0.94) **GBM** (*FOXM1*: *R* = 0.87; *E2F1*: *R* = 0.75; *RAD51*: *R* = 0.87)
**Positive correlation between *UBE2C* and tumor suppressor genes in cancers**			
***BUB1B:* Strong and very strong** (BLCA, BRCA, COAD, ESCA, KICH, KIRC, KIRP, LIHC, LUAD, LUSC, PAAD, READ, SKCM, STAD, UCEC, ACC, DLBC, LAML, UCS, THYM, GBM, HNSC); **Moderate** (PRAD, THCA, OV); **Weak** (LGG) ***BRCA1:* Strong and very strong** (COAD, ESCA, LUAD, LUSC, PAAD, READ, ACC, DLBC, LAML, LGG, GBM); **Moderate** (BLCA, KICH, KIRC, KIRP, LIHC, PRAD, SKCM, STAD, OV, UCS, THYM, HNSC); **Weak** (BRCA and UCEC); **Very weak** (TGCT) ***BRCA2:* Strong and very strong** (COAD, ESCA, READ, DLBC, LAML, LGG, GBM); **Moderate** (BLCA, BRCA, KIRP, LIHC, LUAD, LUSC, PAAD, SKCM, STAD, UCEC, ACC, OV, and UCS); **Weak** (KICH, KIRC, PRAD, THCA, HNSC); **Very weak** (THYM) ***CHK2:* Strong and very strong** (COAD, LUSC, PAAD, READ, UCEC, DLBC, LAML, LGG, GBM, TGCT); **Moderate** (BLCA, BRCA, ESCA, KIRC, LUAD, SKCM, STAD, UCS, HNSC); **Weak** (LIHC, PRAD, THCA, ACC); **Very weak** (KIRP and OV) ***TP53:* Strong and very strong** (ESCA, PAAD, READ, DLBC, LAML, THYM, LGG, GBM, TGCT); **Moderate** (COAD, LIHC, LUSC, UCS); **Weak** (KIRP, LUAD, STAD, THCA, UCEC, ACC); **Very weak** (BLCA, BRCA, KIRC, PRAD, SKCM, OV, HNSC) ***ATM*: Weak** (DLBC); **Very weak** (ESCA, KICH, KIRP, LIHC, PRAD, SKCM, DLBC, LGG) ***ATR*: Strong and very strong** (DLBC and LAML); **Moderate** (ESCA, PAAD, THYM); **Weak** (BLCA, COAD, KICH, KIRC, STAD, HNSC); **Very weak** (BRCA, KIRP, LIHC, LUSC, PRAD, READ, LGG, GBM) ***CYLD*: Weak** (PAAD, DLBC); **Very weak** (BLCA, BRCA, ESCA, KICH, LIHC, LGG, HNSC)			

More details are given in Supplementary Table S1.

**Table 3:** Statistically significant overexpression of different proposed target genes in most common cancers.

Genes	Cancers with Overexpression of Proposed Cancer Target Genes (Normal-vs.-Primary)	OS (Higher Expression Levels-vs.-Lower Expression Levels)
***MTFR2 (FAM54A)***	**BLCA** (*p*-value < 10^−16^), **BRCA** (*p*-value < 10^−12^), **Metastatic breast cancer** (MYC-amplification (+) vs. MYC-amplification (-): *p*-value < 10^−12^, CCND1-amplification (+) vs. CCND1-amplification (−): *p*-value < 10^−12^, ERBB2-amplification (+) vs. ERBB2-amplification (−): *p*-value < 10^−12^), **CESC** (*p*-value < 10^−12^), **COAD** (*p*-value < 10^−12^), **ESCA** (*p*-value < 10^−12^), **GBM** (*p*-value < 10^−12^), **HNSC** (*p*-value < 10^−12^), **KICH** (*p*-value = 0.017), KIRC (*p*-value < 10^−12^), **KIRP** (*p*-value < 10^−12^), LIHC (*p*-value < 10^−12^), **LUAD** (*p*-value < 10^−12^), LUSC (*p*-value < 10^−12^), **PRAD** (*p*-value < 10^−5^, Molecular subtypes of PRAD: Normal-vs.-ERG fusion: *p*-value < 10^−3,^ Normal-vs.-ETV1 fusion: *p*-value < 10^−3^, Normal-vs.-SPOP mutation: *p*-value < 10^−3^), **Metastatic prostate cancer** (ERG-fusion (+) vs. ERG-fusion (-): *p*-value < 10^−12^, AR-amplification (+) vs. AR-amplification (−): *p*-value < 10^−12^), **READ** (*p*-value < 10^−6^), **STAD** (*p*-value < 10^−12^), **UCEC** (*p*-value < 10^−12^).	**ACC** (*p*-value < 0.0001) **LGG** (*p*-value < 0.0001) **KICH** (*p*-value < 0.0001) **KIRC** (*p*-value < 0.0001) **KIRP** (*p*-value < 0.0001) **LIHC** (*p*-value = 0.00018) **UCEC** (*p*-value = 0.043)
***MND1***	**BLCA** (*p*-value < 10^−6^), **BRCA** (*p*-value < 10^−12^), **COAD** (*p*-value < 10^−12^), **ESCA** (*p*-value < 10^−12^), **CESC** (*p*-value = *p*-value < 10^−12^), **HNSC** (*p*-value < 10^−12^), **KICH** (*p*-value < 10^−9^), **KIRC** (*p*-value < 10^−12^), **KIRP** (Normal-vs.-Primary: *p*-value < 10^−15^), **LIHC** (*p*-value < 10^−12^), **LUAD** (*p*-value < 10^−12^), **LUSC** (*p*-value < 10^−12^), **PRAD** (*p*-value < 10^−8^, Molecular subtypes of PRAD: Normal-vs.-ERG fusion: *p*-value < 10^−4,^ Normal-vs.-ETV1 fusion: *p*-value < 10^−3^, Normal-vs.-ETV4 fusion: *p*-value < 10^−4^), **READ** (*p*-value < 10^−8^), **STAD** (*p*-value < 10^−12^), **THCA** (*p*-value = 0.0348), **UCEC** (*p*-value < 10^−12^).	**ACC** (*p*-value = 0.016) **LGG** (*p*-value < 0.0001) **KICH** (*p*-value = 0.00025) **KIRC** (*p*-value = 0.046) **KIRP** (*p*-value < 0.0001) **LIHC** (*p*-value = 0.0054) **LUAD** (*p*-value = 0.0034) **PAAD** (*p*-value = 0.048) **SKCM** (*p*-value = 0.047)
***FAM72D***	**BLCA** (*p*-value < 10^−12^), **BRCA** (*p*-value < 10^−12^), **Metastatic breast cancer** (amplification (+) vs. ERBB2-amplification (−): *p*-value < 10^−2^), **COAD** (*p*-value < 10^−12^), **ESCA** (*p*-value < 10^−12^), **GBM** (*p*-value < 10^−12^), **HNSC** (*p*-value < 10^−12^), **KIRC** (*p*-value < 10^−12^), **KIRP** (*p*-value < 10^−13^), **LIHC** (*p*-value < 10^−12^), **LUAD** (*p*-value < 10^−12^), **LUSC** (*p*-value < 10^−12^), **PRAD** (*p*-value < 10^−10^, Molecular subtypes of PRAD: Normal-vs.-ERG fusion: *p*-value < 10^−7,^ Normal-vs.-ETV1 fusion: *p*-value < 10^−3^, Normal-vs.-SPOP mutation: *p*-value < 10^−5^), **READ** (*p*-value < 10^−11^), **STAD** (*p*-value < 10^−12^), **UCEC** (*p*-value < 10^−12^).	**ACC** (*p*-value < 0.0001) **LGG** (*p*-value < 0.0001) **BRCA** (*p*-value = 0.041) **KICH** (*p*-value = 0.0012) **KIRC** (*p*-value < 0.0001) **KIRP** (*p*-value < 0.0001) **LIHC** (*p*-value = 0.0024) **LUAD** (*p*-value = 0.009)
***POC1A***	**BLCA** (*p*-value < 10^−10^), **BRCA** (*p*-value < 10^−12^), **Metastatic breast cancer** (amplification (+) vs. ERBB2-amplification (−): *p*-value: 0.0279), **CESC** (*p*-value < 10^−9^), **COAD** (*p*-value < 10^−12^), **ESCA** (*p*-value < 10^−11^), **GBM** (*p*-value < 10^−12^), **HNSC** (*p*-value < 10^−12^), **KICH** (*p*-value: 0.016), **KIRP** (*p*-value < 10^−5^), **LIHC** (*p*-value < 10^−12^), **LUAD** (*p*-value < 10^−12^), **LUSC** (*p*-value < 10^−12^), **PRAD** (*p*-value < 10^−15^, Molecular subtypes of PRAD: Normal-vs.-ERG fusion: *p*-value < 10^−7,^ Normal-vs.-ETV1 fusion: *p*-value < 10^−3^, Normal-vs.-ETV4 fusion: *p*-value < 10^−2^, Normal-vs.-SPOP mutation: *p*-value < 10^−10^), **READ** (*p*-value < 10^−5^), **STAD** (*p*-value < 10^−16^), **THCA** (*p*-value < 10^−8^), **UCEC** (*p*-value < 10^−12^).	**ACC** (*p*-value < 0.0001) **LGG** (*p*-value < 0.0001) **KICH** (*p*-value = 0.00017) **KIRC** (*p*-value < 0.0001) **KIRP** (*p*-value < 0.0001) **LIHC** (*p*-value = 0.028) **LUAD** (*p*-value = 0.0094) **OV** (*p*-value = 0.039) **PAAD** (*p*-value = 0.012) **SKCM** (*p*-value = 0.0039) **STAD** (*p*-value = 0.013)

**Table 4:** Biological pathways related to proteins involved in UBE2C network.

KEGG Number	Cellular Process	Protein Gene
hsa04110	Cell cycle	ATM, ATR, BUB1, BUB1B, CCNA2, CCNB1, CCNB2, CDC20, CDC25A, CDC25C, CDK1, CHEK1, E2F1, MAD2L1, ORC6, PLK1, TP53, TTK, CHEK2
hsa04114	Oocyte meiosis	AURKA, BUB1, CCNB1, CCNB2, CDC20, CDC25C, CDK1, MAD2L1, PLK1, SGOL1
hsa04914	Progesterone-mediated oocyte maturation	AURKA, BUB1, CCNA2, CCNB1, CCNB2, CDC25A, CDC25C, CDK1, MAD2L1, PLK1
hsa04218	Cellular senescence	ATM, ATR, CCNA2, CCNB1, CCNB2, CCNE1, CCNE2, CDC25A, CDK1, CHEK1, CHEK2, E2F1, FOXM1, MYBL2, TP53
hsa04115	p53 signaling pathway	ATM, ATR, CCNB1, CCNB2, CDK1, CDKN1A, CHEK1, CHEK2, GTSE1, MDM2, TP53
hsa04120	Ubiquitin mediated proteolysis	BRCA1, CDC20, UBE2C, UBE2S
hsa04068	FoxO signaling pathway	ATM, CCNB1, CCNB2, CDKN1B, PLK1, PLK4
hsa03030	DNA replication	FEN1, RNASEH2A
hsa03440	Homologous recombination	ATM, BRCA1, BRCA2, RAD54L
hsa01522	Endocrine resistance	E2F1, TP53
hsa03460	Fanconi anemia pathway	ATR, BRCA1, BRCA2, UBE2T
hsa05200	Pathways in cancer	BRCA2, CKS2, E2F1, TP53
hsa04151	PI3K-Akt signaling pathway	BRCA1, TP53
hsa05202	Transcriptional misregulation in cancer	ATM, TP53
hsa03410	Base excision repair	FEN1
hsa05222	Small cell lung cancer	CKS2, E2F1, TP53
hsa05215	Prostate cancer	E2F1, TP53
hsa05226	Gastric cancer	E2F1, TP53
hsa05220	Chronic myeloid leukemia	E2F1, TP53
hsa05219	Bladder cancer	E2F1, TP53
hsa05214	Glioma	E2F1, TP53
hsa05218	Melanoma	E2F1, TP53
hsa05212	Pancreatic cancer	BRCA2, E2F1, TP53
hsa05224	Breast cancer	BRCA1, BRCA2, E2F1, TP53
hsa05223	Non-small cell lung cancer	E2F1, TP53

## Supplementary Materials

The following are available online at https://www.mdpi.com/1422-0067/20/9/2228/s1.
