# Supplementary material for: A Comprehensive Bioinformatics Analysis of UBE2C in Cancers
Source: Int J Mol Sci. 2019 May 7;20(9):2228. doi: 10.3390/ijms20092228 (PMC6539744; doi:10.3390/ijms20092228)
Supplement: Supplementary file 1 [file ijms-20-02228-s001.zip › ijms-486730 supplementary final/Figure S2 - S2-UBE2C Expression in different subtypes of cancers.pdf]

Panel 1. *UBE2C* expression in urologic tumours.

A

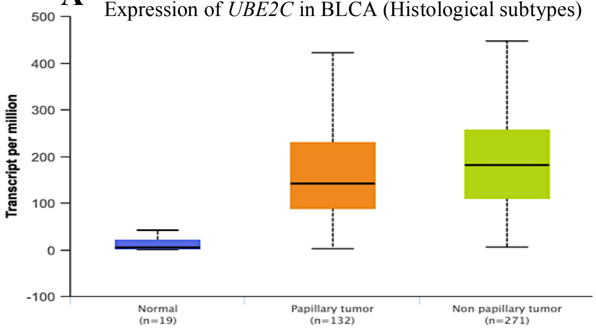

Comparison	p-value
Normal-vs-Papillary tumors	$<10^{-12}$
Normal-vs-NonPapillary tumors	$<10^{-12}$
Papillary tumors-vs-NonPapillary tumors	0.014

B

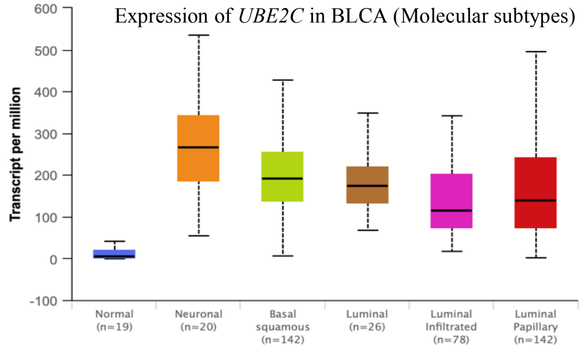

Comparison	p-value
Normal-vs-Neuronal	$<10^{-8}$
Normal-vs-Basal squamous	$<10^{-12}$
Normal-vs-Luminal	$<10^{-10}$
Normal-vs-Luminal_Infiltrated	$<10^{-9}$
Normal-vs-Luminal_Papillary	$<10^{-12}$
Neuronal-vs-Basal squamous	0.02
Neuronal-vs-Luminal	0.03
Neuronal-vs-Luminal_Infiltrated	0.014
Neuronal-vs-Luminal_Papillary	$<10^{-3}$
Basal squamous-vs-Luminal	0.49
Basal squamous-vs-Luminal_Infiltrated	0.032
Basal squamous-vs-Luminal_Papillary	$<10^{-3}$
Luminal-vs-Luminal_Infiltrated	0.22
Luminal-vs-Luminal_Papillary	0.42
Luminal_Infiltrated-vs-Luminal_Papillary	0.61

C

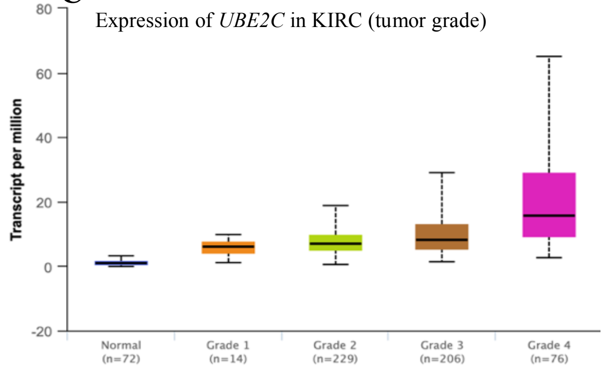

Comparison	p-value
Normal-vs-Grade 1	$<10^{-3}$
Normal-vs-Grade 2	$<10^{-11}$
Normal-vs-Grade 3	$<10^{-9}$
Normal-vs-Grade 4	$<10^{-6}$
Grade 1-vs-Grade 2	$<10^{-4}$
Grade 1-vs-Grade 3	$<10^{-6}$
Grade 1-vs-Grade 4	$<10^{-5}$
Grade 2-vs-Grade 3	$<10^{-3}$
Grade 2-vs-Grade 4	$<10^{-5}$
Grade 3-vs-Grade 4	$<10^{-3}$

D

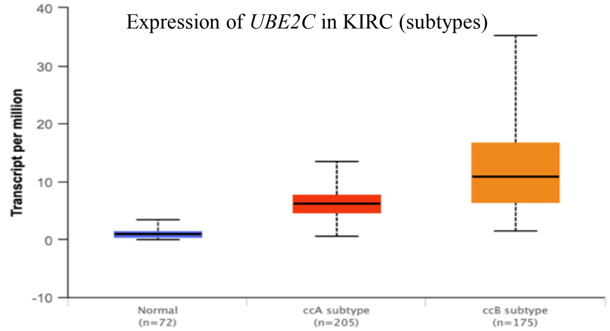

Comparison	p-value
Normal-vs-ccA subtype	10^{-8}
Normal-vs-ccB subtype	$<10^{-8}$
ccA subtype-vs-ccB subtype	$<10^{-5}$

E

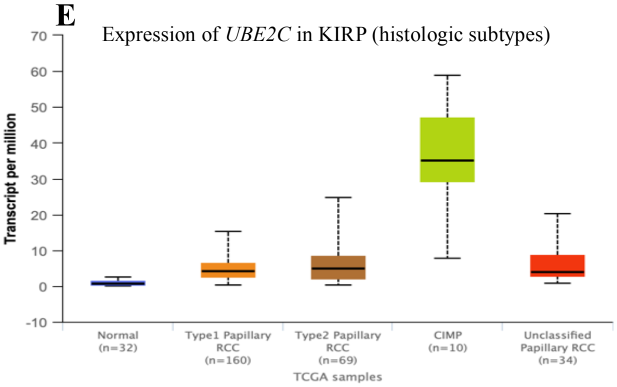

Comparison	p-value
**Normal-VS-Type1 PRCC**	**$<10^{-12}$**
**Normal-VS-Type2 PRCC**	**$<10^{-7}$**
**Normal-VS-KIRP CIMP**	**$<10^{-3}$**
**Normal-VS-Unclassified PRCC**	**$<10^{-3}$**
**Type1 PRCC-VS-Type2 PRCC**	**$<10^{-3}$**
**Type1 PRCC-VS-KIRP CIMP**	**$<10^{-3}$**
Type1 PRCC-VS-Unclassified PRCC	0.095
**Type2 PRCC-VS-KIRP CIMP**	**0.015**
Type2 PRCC-VS-Unclassified PRCC	0.89
**KIRP CIMP-VS-Unclassified PRCC**	**0.015**

F

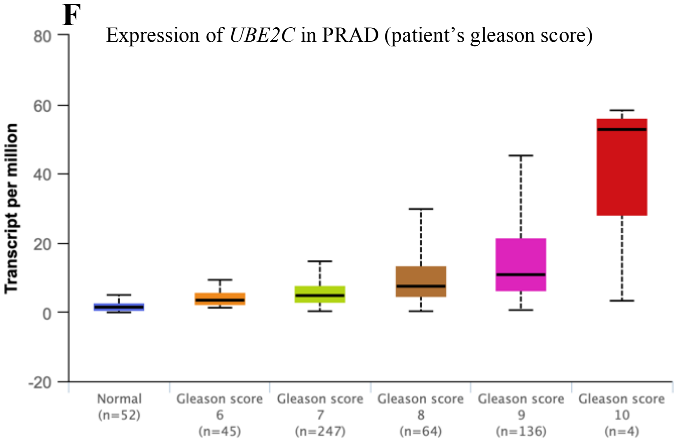

Comparison	p-value
Normal-vs-Gleason score 6	0.53
**Normal-vs-Gleason score 7**	**$<10^{-3}$**
**Normal-vs-Gleason score 8**	**$<10^{-5}$**
**Normal-vs-Gleason score 9**	**$<10^{-4}$**
Normal-vs-Gleason score 10	0.16
**Gleason score 6-vs-Gleason score 7**	**$<10^{-5}$**
**Gleason score 6-vs-Gleason score 8**	**$<10^{-5}$**
**Gleason score 6-vs-Gleason score 9**	**$<10^{-4}$**
Gleason score 6-vs-Gleason score 10	0.16
**Gleason score 7-vs-Gleason score 8**	**$<10^{-3}$**
**Gleason score 7-vs-Gleason score 9**	**$<10^{-3}$**
Gleason score 7-vs-Gleason score 10	0.17
Gleason score 8-vs-Gleason score 9	0.058
Gleason score 8-vs-Gleason score 10	0.2
Gleason score 9-vs-Gleason score 10	0.1

G

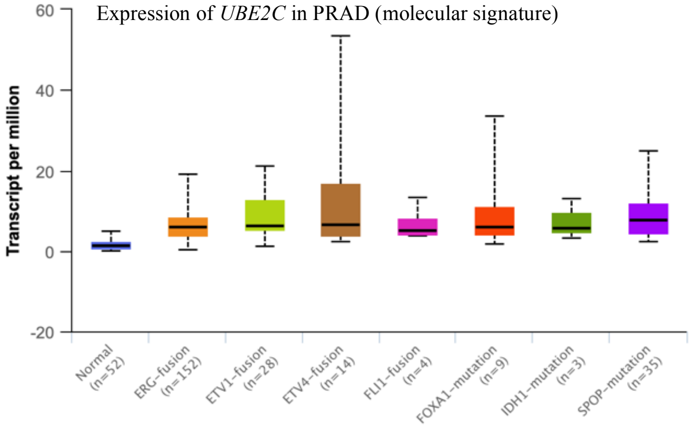

Comparison	p-value
**Normal-vs-ERG fusion**	**$<10^{-5}$**
**Normal-vs-ETV1 fusion**	**$<10^{-3}$**
Normal-vs-ETV4 fusion	0.11
Normal-vs-FLI1 fusion	0.4
Normal-vs-FOXA1 mutation	0.08
Normal-vs-IDH1 mutation	0.4
**Normal-vs-SPOP mutation**	**$<10^{-5}$**
ERG fusion-vs-ETV1 fusion	0.62
ERG fusion-vs-ETV4 fusion	0.21
ERG fusion-vs-FLI1 fusion	0.62
ERG fusion-vs-FOXA1 mutation	0.34
ERG fusion-vs-IDH1 mutation	0.75
ERG fusion-vs-SPOP mutation	0.1
ETV1 fusion-vs-ETV4 fusion	0.23
ETV1 fusion-vs-FLI1 fusion	0.48
ETV1 fusion-vs-FOXA1 mutation	0.44
ETV1 fusion-vs-IDH1 mutation	0.61
ETV1 fusion-vs-SPOP mutation	0.44
ETV4 fusion-vs-FLI1 fusion	0.17
ETV4 fusion-vs-FOXA1 mutation	0.39
ETV4 fusion-vs-IDH1 mutation	0.18
ETV4 fusion-vs-SPOP mutation	0.28
FLI1 fusion-vs-FOXA1 mutation	0.39
FLI1 fusion-vs-IDH1 mutation	0.89
FLI1 fusion-vs-SPOP mutation	0.37
FOXA1 mutation-vs-IDH1 mutation	0.49
FOXA1 mutation-vs-SPOP mutation	0.54
IDH1 mutation-vs-SPOP mutation	0.49

H

Expression of *UBE2C* in MET500 PRAD
(*AR* amplification status)

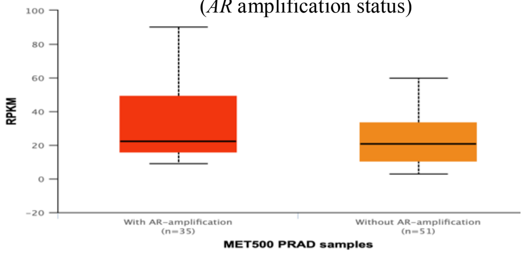

Comparison	p-value
AR-amplification(+) vs AR-amplification(-)	0.52

Expression of *UBE2C* in MET500 PRAD
(*ERG* fusion status)

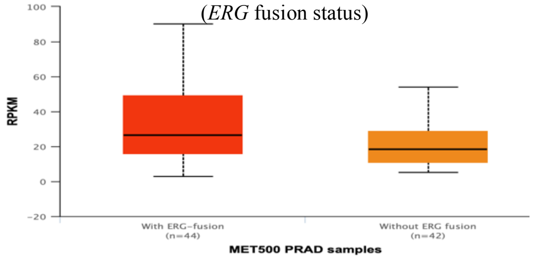

Comparison	p-value
ERG-fusion(+) vs ERG-fusion(-)	0.11

Panel 2. *UBE2C* expression in BRCA and metastatic BRCA

A

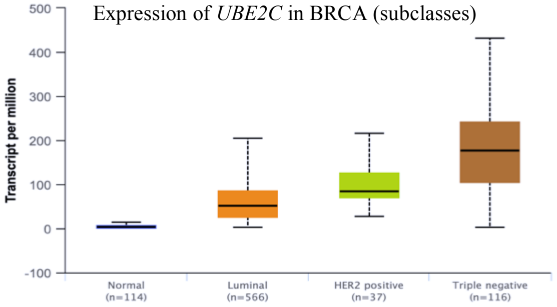

Comparison	p-value
Normal-vs-Luminal	$<10^{-12}$
Normal-vs-HER2 Positive	$<10^{-10}$
Normal-vs-TNBC	$<10^{-12}$
Luminal-vs-HER2 Positive	0.02
Luminal-vs-TNBC	$<10^{-12}$
HER2 Positive-vs-TNBC	$<10^{-6}$

B

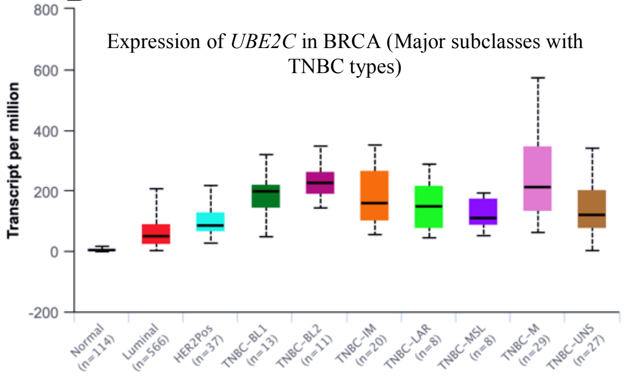

Comparison (only statistically significant values are shown)	p-value
Normal-vs-Luminal	$<10^{-12}$
Normal-vs-HER2Pos	$<10^{-10}$
Normal-vs-TNBC-BL1	$<10^{-7}$
Normal-vs-TNBC-BL2	$<10^{-8}$
Normal-vs-TNBC-IM	$<10^{-8}$
Normal-vs-TNBC-LAR	$<10^{-3}$
Normal-vs-TNBC-MSL	$<10^{-4}$
Normal-vs-TNBC-M	$<10^{-10}$
Normal-vs-TNBC-UNS	$<10^{-5}$
Luminal-vs-HER2Pos	0.02
Luminal-vs-TNBC-BL1	$<10^{-7}$
Luminal-vs-TNBC-BL2	$<10^{-10}$
Luminal-vs-TNBC-IM	$<10^{-8}$
Luminal-vs-TNBC-LAR	$<10^{-3}$
Luminal-vs-TNBC-M	$<10^{-7}$
Luminal-vs-TNBC-UNS	$<10^{-3}$
HER2Pos-vs-TNBC-BL1	$<10^{-3}$
HER2Pos-vs-TNBC-BL2	$<10^{-5}$
HER2Pos-vs-TNBC-IM	$<10^{-3}$
HER2Pos-vs-TNBC-M	$<10^{-5}$
TNBC-BL1-vs-TNBC-MSL	0.031
TNBC-BL2-vs-TNBC-LAR	0.044
TNBC-BL2-vs-TNBC-MSL	$<10^{-4}$
TNBC-MSL-vs-TNBC-M	$<10^{-4}$

C

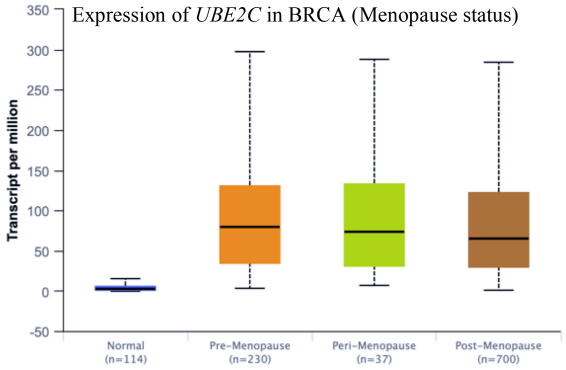

Comparison	p-value
Normal-vs-Pre-Menopause	$<10^{-12}$
Normal-vs-Peri-Menopause	$<10^{-6}$
Normal-vs-Post-Menopause	$<10^{-12}$
Pre-Menopause-vs-Peri-Menopause	0.44
Pre-Menopause-vs-Post-Menopause	0.16
Peri-Menopause-vs-Post-Menopause	0.21

D

Expression of *UBE2C* in BRCA (Histologic subtypes)

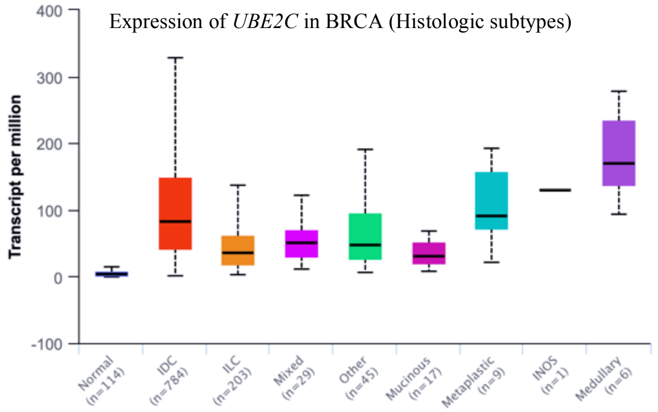

Comparison (statistically non-significant values are not shown)	p-value
**Normal-vs-IDC**	**$<10^{-12}$**
**Normal-vs-ILC**	**$<10^{-12}$**
**Normal-vs-Mixed**	**$<10^{-6}$**
**Normal-vs-Other**	**$<10^{-7}$**
**Normal-vs-Mucinous**	**$<10^{-3}$**
**Normal-vs-Metaplastic**	**$<10^{-3}$**
Normal-vs-INOS	N/A
**Normal-vs-Medullary**	**$<10^{-3}$**
**IDC-vs-ILC**	**$<10^{-12}$**
**IDC-vs-Mixed**	**$<10^{-4}$**
**IDC-vs-Other**	**$<10^{-3}$**
**IDC-vs-Mucinous**	**$<10^{-4}$**
IDC-vs-INOS	N/A
**ILC-vs-Other**	**0.036**
**ILC-vs-Metaplastic**	**$<10^{-3}$**
ILC-vs-INOS	N/A
**ILC-vs-Medullary**	**$<10^{-10}$**
Mixed-vs-INOS	N/A
**Mixed-vs-Medullary**	**$<10^{-4}$**
Other-vs-INOS	N/A
**Other-vs-Medullary**	**$<10^{-3}$**
**Mucinous-vs-Metaplastic**	**0.032**
Mucinous-vs-INOS	N/A
**Mucinous-vs-Medullary**	**$<10^{-5}$**
Metaplastic-vs-INOS	N/A
**Metaplastic-vs-Medullary**	**0.035**
INOS-vs-Medullary	N/A

E

Expression of *UBE2C* in MET500 BRCA (*MYC* amplification status)

Expression of *UBE2C* in MET500 BRCA (*CCND1* amplification status)

Expression of *UBE2C* in MET500 BRCA (*ERBB2* amplification status)

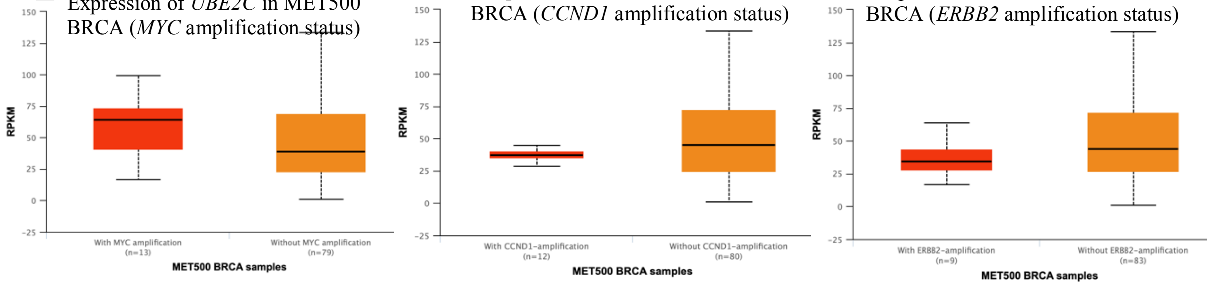

Comparison	p-value
MYC-amplification(+) vs MYC-amplification(-)	0.32

Comparison	p-value
CCND1-amplification (+) vs CCND1-amplification(-)	0.05

Comparison	p-value
ERBB2-amplification (+) vs ERBB2-amplification(-)	0.11

Panel 3. *UBE2C* expression in digestive system tumours

A Expression of *UBE2C* in COAD (Histologic subtypes)

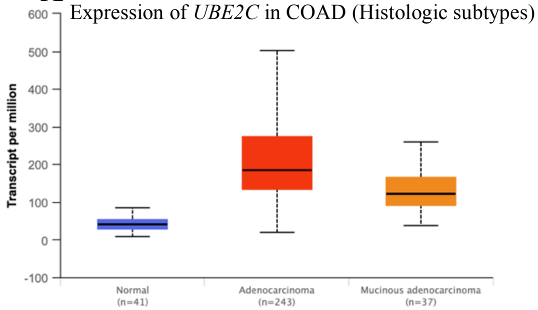

Comparison	p-value
**Normal-vs-Adenocarcinoma**	**$<10^{-12}$**
**Normal-vs-Mucinous-adenocarcinoma**	**$<10^{-11}$**
**Adenocarcinoma-vs-Mucinous-adenocarcinoma**	**$<10^{-10}$**

B Expression of *UBE2C* in ESCA (tumor histology)

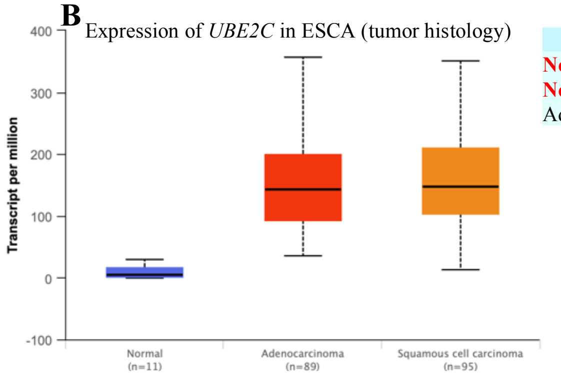

Comparison	p-value
**Normal-vs-Adenocarcinoma**	**$<10^{-12}$**
**Normal-vs-Squamous-cell-carcinoma**	**$<10^{-12}$**
Adenocarcinoma-vs-Squamous-cell-carcinoma	0.55

C Expression of *UBE2C* in HNSC (tumor grade)

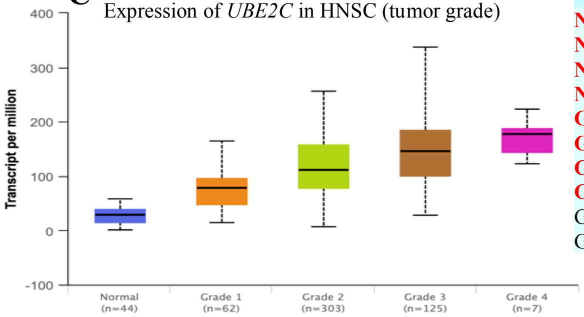

Comparison	p-value
**Normal-vs-Grade 1**	**$<10^{-12}$**
**Normal-vs-Grade 2**	**$<10^{-12}$**
**Normal-vs-Grade 3**	**$<10^{-12}$**
**Normal-vs-Grade 4**	**$<10^{-5}$**
**Grade 1-vs-Grade 2**	**$<10^{-5}$**
**Grade 1-vs-Grade 3**	**$<10^{-10}$**
**Grade 1-vs-Grade 4**	**$<10^{-4}$**
**Grade 2-vs-Grade 3**	**$<10^{-5}$**
Grade 2-vs-Grade 4	0.096
Grade 3-vs-Grade 4	0.64

D Expression of *UBE2C* in HNSC (HPV status)

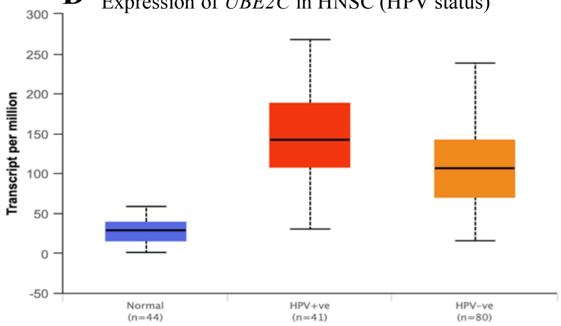

Comparison	p-value
**Normal-vs-HPV+ve**	**$<10^{-11}$**
**Normal-vs-HPV-ve**	**$<10^{-12}$**
**HPV+ve-vs-HPV-ve**	**$<10^{-3}$**

E

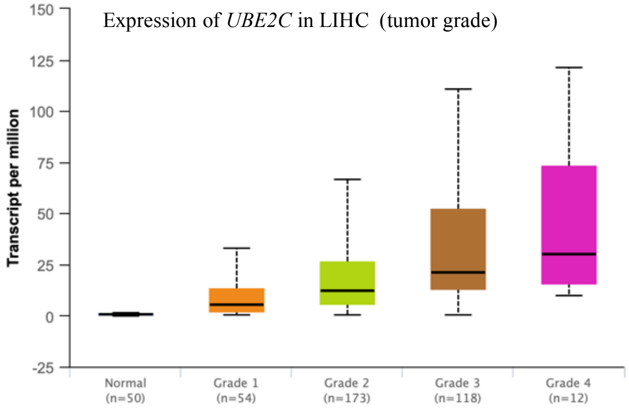

Comparison	p-value
**Normal-vs-Grade 1**	**$<10^{-5}$**
**Normal-vs-Grade 2**	**$<10^{-12}$**
**Normal-vs-Grade 3**	**$<10^{-12}$**
**Normal-vs-Grade 4**	**$<10^{-3}$**
Grade 1-vs-Grade 2	0.13
**Grade 1-vs-Grade 3**	**$<10^{-4}$**
**Grade 1-vs-Grade 4**	**0.02**
**Grade 2-vs-Grade 3**	**$<10^{-3}$**
Grade 2-vs-Grade 4	0.16
Grade 3-vs-Grade 4	0.96

F

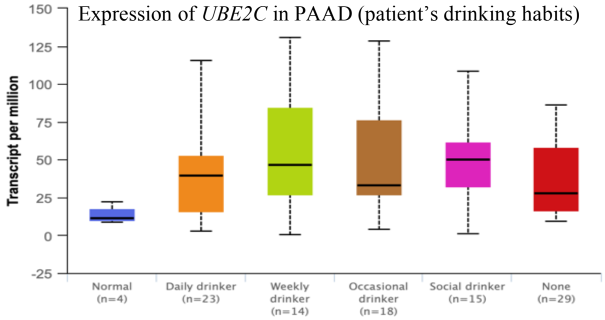

Comparison (only statistically significant values are shown)	p-value
**Normal-vs-Weekly Drinker**	**0.025**
**Normal-vs-Occasional Drinker**	**$<10^{-3}$**

G

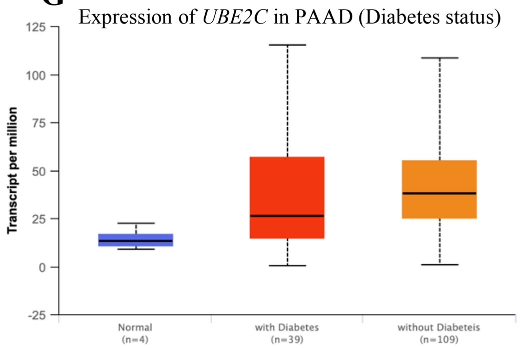

Comparison	p-value
**Normal-vs-Diabetic**	**$<10^{-4}$**
**Normal-vs-NonDiabetic**	**$<10^{-7}$**
Diabetic-vs-NonDiabetic	0.37

H

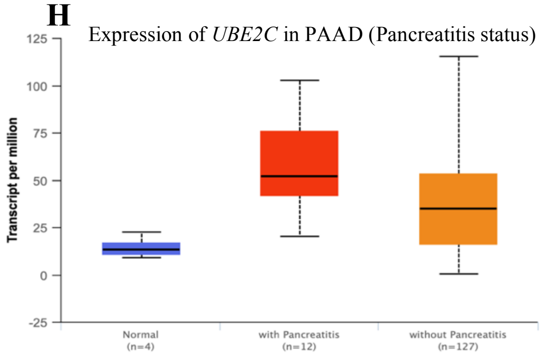

Comparison	p-value
**Normal-vs-Pancreatitis**	**$<10^{-4}$**
**Normal-vs-NoPancreatitis**	**$<10^{-7}$**
Pancreatitis-vs-NoPancreatitis	0.24

I

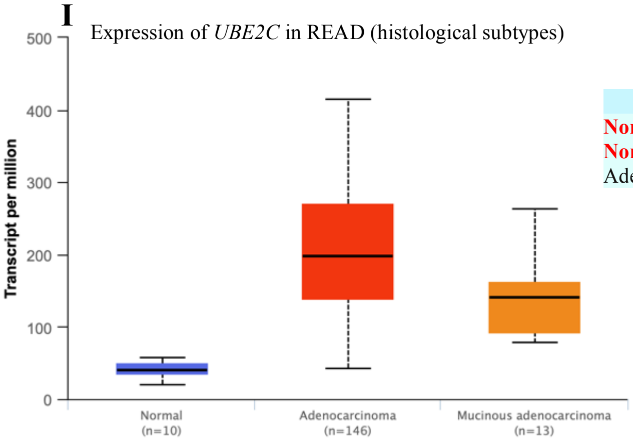

Comparison	p-value
**Normal-vs-Adenocarcinoma**	**$<10^{-12}$**
**Normal-vs-Mucinous-adenocarcinoma**	**$<10^{-3}$**
Adenocarcinoma-vs-Mucinous-adenocarcinoma	0.11

J

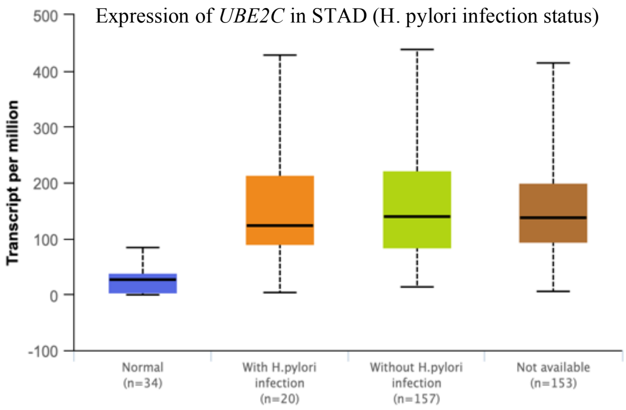

Comparison	p-value
**Normal-vs-Tumors(with H.pylori infection)**	**$<10^{-4}$**
**Normal-vs-Tumors(without H.pylori infection)**	**$<10^{-12}$**
**Normal-vs-Tumors(Not available)**	**$<10^{-12}$**
Tumors(with H.pylori infection)-vs-Tumors(without H.pylori infection)	0.87
Tumors(with H.pylori infection)-vs-Tumors(Not available)	0.8
Tumors(without H.pylori infection)-vs-Tumors(Not available)	0.39

K

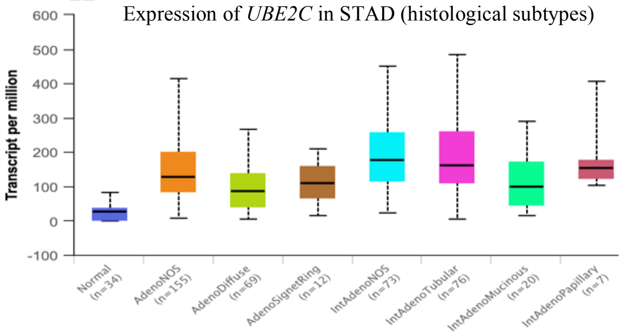

Comparison (only statistically significant values are shown)	p-value
**Normal-vs-Adenocarcinoma(NOS)**	**$<10^{-12}$**
**Normal-vs-Adenocarcinoma(Diffuse)**	**$<10^{-10}$**
**Normal-vs-Adenocarcinoma(Signet Ring)**	**$<10^{-3}$**
**Normal-vs-IntestinalAdenocarcinoma(NOS)**	**$<10^{-12}$**
**Normal-vs-IntestinalAdenocarcinoma(Tubular)**	**$<10^{-12}$**
**Normal-vs-IntestinalAdenocarcinoma(Mucinous)**	**$<10^{-4}$**
**Normal-vs-IntestinalAdenocarcinoma(Papillary)**	**0.017**
**Adenocarcinoma(NOS)-vs-Adenocarcinoma(Diffuse)**	**$<10^{-4}$**
**Adenocarcinoma(Diffuse)-vs-IntestinalAdenocarcinoma(NOS)**	**$<10^{-7}$**
**Adenocarcinoma(Diffuse)-vs-IntestinalAdenocarcinoma(Tubular)**	**$<10^{-6}$**
**Adenocarcinoma(Signet Ring)-vs-IntestinalAdenocarcinoma(NOS)**	**0.032**
**IntestinalAdenocarcinoma(NOS)-vs-IntestinalAdenocarcinoma(Mucinous)**	**0.019**
**IntestinalAdenocarcinoma(Tubular)-vs-IntestinalAdenocarcinoma(Mucinous)**	**0.039**

Panel 4. *UBE2C* expression in lung tumours

A

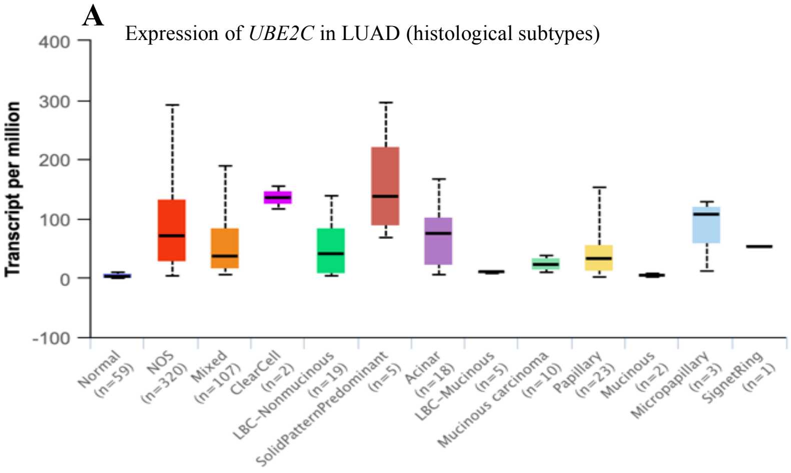

Comparison (only significant changes are shown)	p-value
Normal-vs-NOS	$<10^{-12}$
Normal-vs-Mixed	$<10^{-14}$
Normal-vs-LBC-NonMucinous	$<10^{-4}$
Normal-vs-SolidPatternPredominant	0.019
Normal-vs-Acinar	$<10^{-5}$
Normal-vs-LBC-Mucinous	$<10^{-5}$
Normal-vs-Mucinous carcinoma	$<10^{-3}$
Normal-vs-Papillary	$<10^{-4}$
NOS-vs-Mixed	$<10^{-5}$
NOS-vs-LBC-NonMucinous	$<10^{-4}$
NOS-vs-Acinar	0.017
NOS-vs-LBC-Mucinous	$<10^{-12}$
NOS-vs-Mucinous carcinoma	$<10^{-6}$
NOS-vs-Papillary	0.032
Mixed-vs-SolidPatternPredominant	$<10^{-3}$
Mixed-vs-LBC-Mucinous	$<10^{-11}$
Mixed-vs-Mucinous carcinoma	0.026
ClearCell-vs-Mucinous carcinoma	$<10^{-3}$
ClearCell-vs-Mucinous	0.021
LBC-NonMucinous-vs-SolidPatternPredominant	$<10^{-3}$
LBC-NonMucinous-vs-LBC-Mucinous	$<10^{-3}$
SolidPatternPredominant-vs-Acinar	0.011
SolidPatternPredominant-vs-LBC-Mucinous	0.023
SolidPatternPredominant-vs-Mucinous carcinoma	0.044
SolidPatternPredominant-vs-Papillary	0.028
Acinar-vs-LBC-Mucinous	$<10^{-4}$
LBC-Mucinous-vs-Mucinous carcinoma	0.028
LBC-Mucinous-vs-Papillary	$<10^{-3}$

B

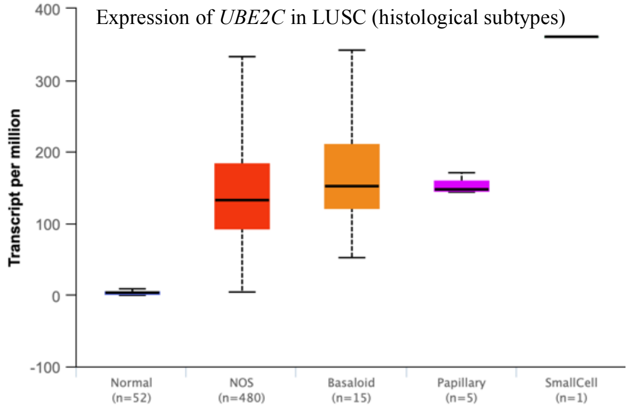

Comparison	p-value
Normal-vs-NOS	$<10^{-12}$
Normal-vs-Basaloid	$<10^{-5}$
Normal-vs-Papillary	0.018
Normal-vs-SmallCell	N/A
NOS-vs-Basaloid	0.059
NOS-vs-Papillary	0.95
NOS-vs-SmallCell	N/A
Basaloid-vs-Papillary	0.43
Basaloid-vs-SmallCell	N/A
Papillary-vs-SmallCell	N/A

Panel 5. *UBE2C* expression in UCEC

A

Expression of *UBE2C* in UCEC (histological subtypes)

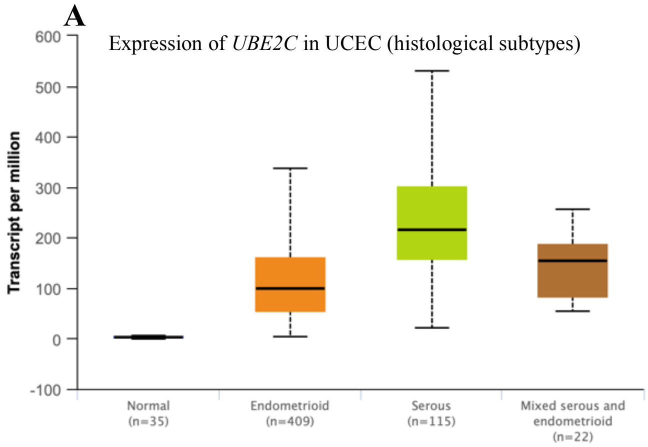

Comparison	p-value
**Normal-vs-Endometrioid**	**$<10^{-12}$**
**Normal-vs-Serous**	**$<10^{-12}$**
**Normal-vs-Mixed serous and endometrioid**	**$<10^{-7}$**
**Endometrioid -vs-Serous**	**$<10^{-13}$**
Endometrioid-vs-Mixed serous and endometrioid	0.16
**Serous-vs-Mixed serous and endometrioid**	**$<10^{-3}$**

B

Expression of *UBE2C* in UCEC (Menopause status)

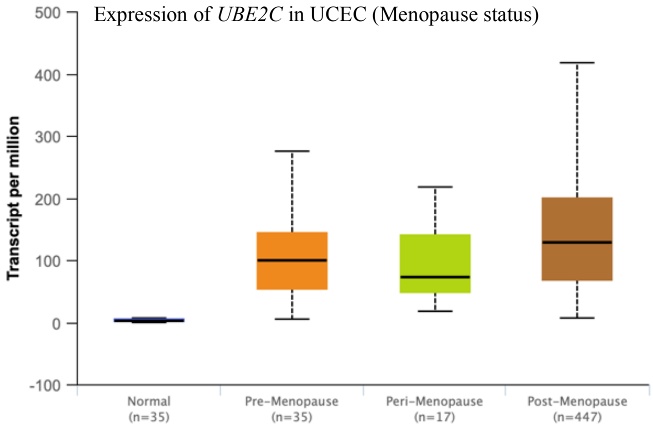

Comparison	p-value
**Normal-vs-Pre-Menopause**	**$<10^{-9}$**
**Normal-vs-Peri-Menopause**	**$<10^{-4}$**
**Normal-vs-Post-Menopause**	**$<10^{-12}$**
Pre-Menopause-vs-Peri-Menopause	0.61
**Pre-Menopause-vs-Post-Menopause**	**$<10^{-3}$**
**Peri-Menopause-vs-Post-Menopause**	**$<10^{-3}$**

Panel 6. *UBE2C* expression based on smoking habits in BLCA, ESCA, LUAD, LUSC

A

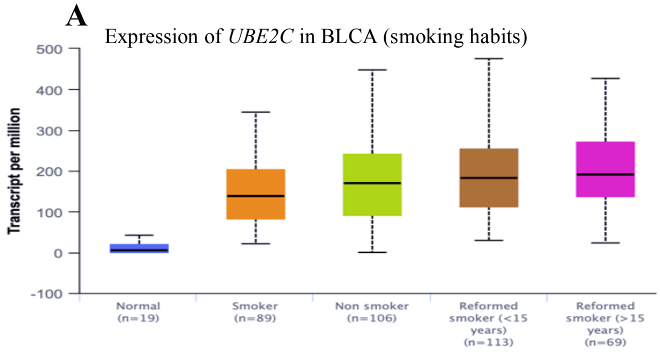

Comparison	p-value
**Normal-vs-Non smoker**	**$<10^{-12}$**
**Normal-vs-Smoker**	**$<10^{-12}$**
**Normal-vs-Reformed smoker1**	**$<10^{-12}$**
**Normal-vs-Reformed smoker2**	**$<10^{-13}$**
Non smoker-vs-Smoker	0.3
Non smoker-vs-Reformed smoker1	0.066
**Non smoker-vs-Reformed smoker2**	**0.017**
Smoker-vs-Reformed smoker1	0.58
Smoker-vs-Reformed smoker2	0.1
Reformed smoker1-vs-Reformed smoker2	0.18

B

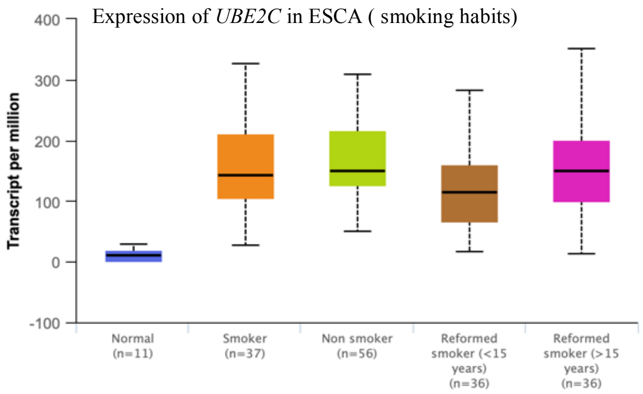

Comparison	p-value
**Normal-vs-Non smoker**	**$<10^{-12}$**
**Normal-vs-Smoker**	**$<10^{-16}$**
**Normal-vs-Reformed smoker1**	**$<10^{-9}$**
**Normal-vs-Reformed smoker2**	**$<10^{-11}$**
Non smoker-vs-Smoker	0.68
Non smoker-vs-Reformed smoker1	0.3
Non smoker-vs-Reformed smoker2	0.59
Smoker-vs-Reformed smoker1	0.18
Smoker-vs-Reformed smoker2	0.85
Reformed smoker1-vs-Reformed smoker2	0.2

C

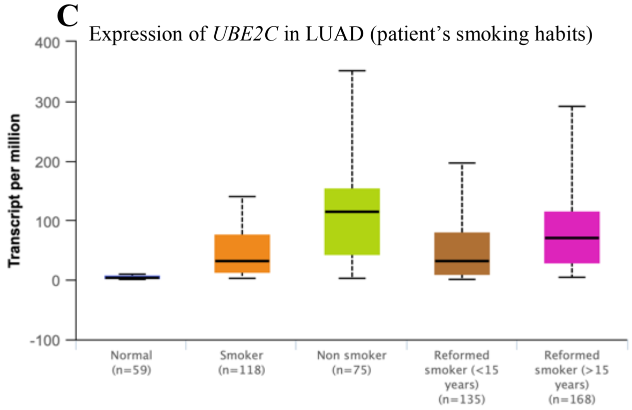

Comparison	p-value
**Normal-vs-Non smoker**	**$<10^{-9}$**
**Normal-vs-Smoker**	**$<10^{-12}$**
**Normal-vs-Reformed smoker1**	**$<10^{-12}$**
**Normal-vs-Reformed smoker2**	**$<10^{-12}$**
**Non smoker-vs-Smoker**	**$<10^{-6}$**
Non smoker-vs-Reformed smoker1	0.96
**Non smoker-vs-Reformed smoker2**	**$<10^{-4}$**
**Smoker-vs-Reformed smoker1**	**$<10^{-7}$**
Smoker-vs-Reformed smoker2	0.16
**Reformed smoker1-vs-Reformed smoker2**	**$<10^{-6}$**

D

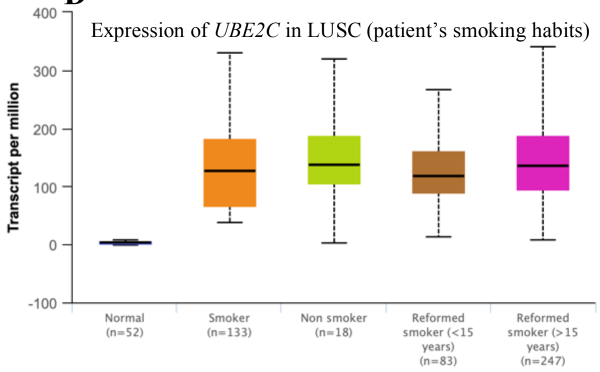

Comparison	p-value
**Normal-vs-Non smoker**	**$<10^{-5}$**
**Normal-vs-Smoker**	**$<10^{-12}$**
**Normal-vs-Reformed smoker1**	**$<10^{-12}$**
**Normal-vs-Reformed smoker2**	**$<10^{-12}$**
Non smoker-vs-Smoker	0.83
Non smoker-vs-Reformed smoker1	0.46
Non smoker-vs-Reformed smoker2	0.86
Smoker-vs-Reformed smoker1	0.14
Smoker-vs-Reformed smoker2	0.25
**Reformed smoker1-vs-Reformed smoker2**	**0.019**
