# Supplementary material for: A Comprehensive Bioinformatics Analysis of UBE2C in Cancers
Source: Int J Mol Sci. 2019 May 7;20(9):2228. doi: 10.3390/ijms20092228 (PMC6539744; doi:10.3390/ijms20092228)
Supplement: Supplementary file 1 [file ijms-20-02228-s001.zip › ijms-486730 supplementary final/Figure S5 - S7-In Silico associations between TSPYL2 and UBE2C.pdf]

The optimal secondary structure upon hybridization is given below. It ranges from positions 327 to 348 in the longer sequence and from positions 189 to 212 in the short sequence.

Total free energy of binding: **−18.82** kcal/mol

Energy from duplex formation: **−43.58** kcal/mol

Opening energy for the longer sequence: **8.82** kcal/mol

Opening energy for the shorter sequence: **15.94** kcal/mol

((((((((((.((((((((((&)))))))))..)).))))))))))

You can access the RNAup output here.

Graphical output

The plot below shows the interaction free energy (RED) ΔG_i and the energy needed to open existing structures in the longer sequence (BLACK) for the target region (top) and the whole range (bottom).

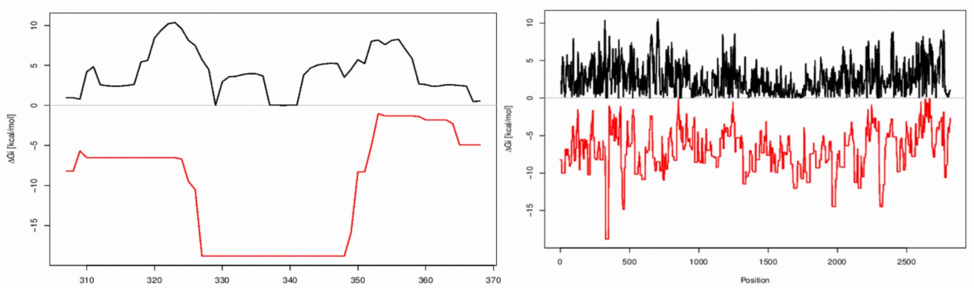

Panel A. RNA-RNA interaction related to TSPYL2 (black) and UBE2C (red).

Protein:
MASQNRDPAATSVAAARKGAEPSGGAARGPVGKRLQQELMTLMMSGDKGISAFPESDNLFKWVGTIHGAAGTVYEDLRYKLSLEFPSGYPYNAPTVKFLTPCYHPNVDTQGNICLDILKEKWSALY
DVRTILLSIQSLLGEPNIDSPLNTHAAELWKNPTAFKKYLQETYSKQVTSQEP

RNA:
UCUCGCUCCACCACUCCUCUCGACCAACGCACUCAGAGGAGUCGAGACGAAUGGCCACGCUGAUCGCCGUCGCUGCGCCGAUUUUCGCUUCCCCGCUCACGCUCAGGGGACUCGACAUGC
UUGCGCCAGCGGUACCUGGCGGGUCUACUCCCCGGAGGCCGGUUCUGGGCGGCGGACUCGUCGAGGCUCAGAGGUGUCGCGCUGGGCGGGGGCGGCGGCGGCGGCGGCGGCGAGGAGGC
UGACGGCGACGGAGGUGGGGUCGUCGCGGGCUCCGAGGUCCUCCUUUGCCUCCGCCGUGUCCACGACCGGCUAUACUCCCCCCACCCUGACCCGGGGCGCGACGGGGGCGGCGGAGGGAU
ACAGUAAGAGCUCCUCCCCCCCUAGGCGCGUAUGAAGUGCGAGCCACGACUCACAGGGCCGACCCUAAGAUGGUAGCUCAGCCCCAUACCCCUCCGCGGGGGCGGGUGCCUCUCGGACCU
UCGUGAGGGGUGAGGACUCCGGAGCCCCCCCUCGGACCUUUAGCUAAAAGUCCAACAUGUCAGCUCGUCAAAACCACCUCUCCCCCGGGAUCUUUGGACAUCGCGUCACCCCACCCGCGG
GGUCUCCAAUCAACUGGGCUUCUCGUUCCUUCUCCGCUAGUAGUAUCACCUCCUACUCCUACUCCUACUCGCCCUCUCAUACUCCUCGUCGUCCUCCGCCGCCGCCGCCGCCUCCUCCUUC
GUCUCCUUCCACUUCUCCCUUUCGUCUCUCUCUUUACGGCUCUCCUACCUCUCGUAGGACGUCCGUGACCUCCUAUAAGUCGACCUAGACCUCCGUCACUUGUAGUUCCGUCCGUUUCGG
AAGGACGCAGAGUUCGCGUUCAAGUAGGUCUACGCUUCUGGGAAGGACCUCGCGUCUCUGGAGUAGUAGGUCGUAUAGGGUCCGAAGACCCAGUUUCGUAAGGAGUUGGUGGGGUCUU
AAAGUUAAAACUAGUUGGCUGCACUACUUCUGUAAAAGGCGAUGAACUGGUUAGACGUCCAUGUCCUAGAGUCUGUAUAGAGGUACCCGAUGUUUUACUUCGACAUGAAGGUCUGAUU
GGGGAUGAAGUGUUUGUACCACUAACAGUUCCUCAAGGUCGCGUUGGCGAGUCCGGCCGACCACAGAGUGAGUUGGGGUUAGGCGACCGUGGCCCCGGUCCUUGGGGUCCGGGCAGCAG
UGCCCUUGGUCCUACGCUCGGUGUCGAAAAAGUCGACCAAGAGUUUGGUAUCGGAGGGUCUCCGACUGUCCUAACGACUCUAAUAGUUCUUACUAGACACCCAAUUGGGAGAUGCGAUG
AUGGACUCUCUUUCCCCGAGGUCCUAUUUCUCUUUCUUCGUUCUUUACUUCUUUGCAUUUUGGUCCCCGUCUACACUCCACCACUAGUACCUUCUGCGGGGACUGAUAAUACGUCACCU
UCUGUAAAAGUCGCUCUAGAGUCUGUAACUACUCUGUUAAGUACUGUAGUUCUAGAGACUGAAGUACCUCUGGUGGCUGAUGAAGCUCUGGUGACUGUUACUCUAUUGACUGUAGUUA
CUCUUGUAGACGCUGUCGCUCUUAGGACUGGUGUUACUCCAGGGGUUGUUGCUCUGGUGACUAUUGUUGCUCUCACGACUACUGGUGCUUUGGUGACUGUUGUUACUCUCACGUCUACU
GUUGUUGCUCUUAGGACUUCUGUUAUUGUUCUUGUGACUACUGUUGCUUCUCUUGGGAUUGUUGUUGCUCUUGUGAAUGCCGUUGUUGAAGAAGUUUCCACCGAAGACCCCGUCGGUA
CCGUUGUUGGUCCUGUCGUCGCUGUCACUGUUACUUCGUCUACUCCGGUCACUACUACUUCUAUUACUACCGUUGCUUCCACUGUUACUCCCGUCACUACUACUACCGUUACUUCCACUG
UUACUUCCGUCGCUACUACUGCUGUCUCUGUAACUCAUGAUACUCUUUCAAUAACUUCUGAAACUGUUCCUAGUCCGACUGAUGCUCCUGCACUAUCUCUAGUAGAGUCUGCUUAGUCA
CCUUCUUCUCCCGUAACUCCUUCCGUAGGUCGUUCUACUCCUGUAGAUACUCCUUCCUUUGAUACUCCUCCUUCCUUCACUUCUACAGACCCUUCUUCCCCUUCUAAGCCUGCUGAGACU
GGAUCUCCUACACGAAGUCCAGGGUUUGCCAACCCGGUUAGGCCCCUUCUCCCCCUUUUGGCCUAUUCCCAAAAGGGGAAAACCCCUAGUGGAGAGACAUAGGGGGUGGGUGAUAGGGU
AAACGGGAGGAGGAGUCGAUCCCGGUGCGCCGGGGUGUAACGUGAAGACCCCCCACUGGCUGAAGCAUGUGCCCAAAUUUCAAAUAAAAAUACCAAAUCAGUAACGUCUCAAGAAUAAA
ACCCCCCUCCCUUUCCCCGAUCAGGGGAAGAAAACCGGGAGGCGGGGGCGUCCGAAGACACACGACGAUUGACAUAAAUAACACUACGGAACCAGUCCCGGGGAGAUGGGUGAAGAGG
GUCAGUCAACACCGGGGUCGGGGAGAGGGACACGACACACCUCACCUGUGGGACUGGGGGCUUCGCCCCUCCCGGCGACACCGGAAGCAGUGUCGGCGCGUCACGGGUACCUCCGCGACG
ACGGUGGAAGGAGAGGGUUCAAGAAAGAGGUAGGGAGAGGAGAAGGGCGGCGCGGCGAUCGGGCGGAGCCACAGAUACGUUCCGGCGAAGCGGUAACGCCAUAAGAAACGCCAUAAGA
ACAGGGGCAGGGGGUCUUCCGAGCGGAGAGGGGCACCUGGGACAAUUAGGGUUAUUUUAAGACUCGUUCAAGU

**Interaction probabilities**	
Prediction using RF classifier	0.9
Prediction using SVM classifier	0.76

RPISeq gives interaction probabilities in the range between 0 and 1. In performance evaluation experiments, predictions with probabilities > 0.5 is considered “positive,” i.e., representing that RNA and protein are likely to interact.

Panel B. Protein-RNA interaction related to UBE2C protein (top) and TSPYL2 RNA (down).

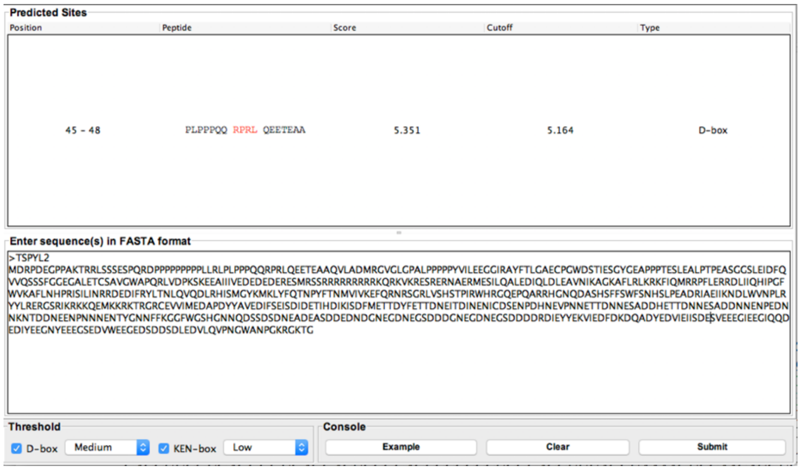

Panel C. The presence of D-box in TSPYL2 protein.
