# Supplementary figures and images for: A Comprehensive Bioinformatics Analysis of UBE2C in Cancers
Source: Int J Mol Sci. 2019 May 7;20(9):2228. doi: 10.3390/ijms20092228 (PMC6539744; doi:10.3390/ijms20092228)

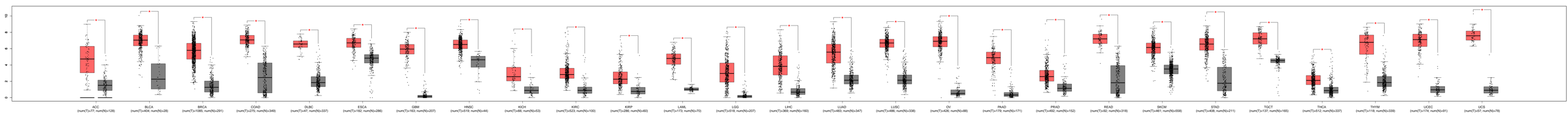

Supplement: Supplementary file 1 [file ijms-20-02228-s001.zip › ijms-486730 supplementary final/Figure S1 - S1-UBE2C Expression_boxplot_.pdf]

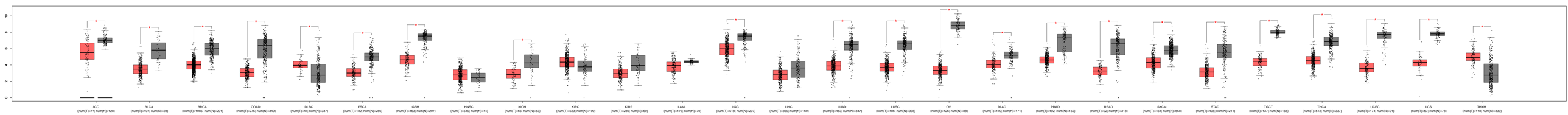

Supplement: Supplementary file 1 [file ijms-20-02228-s001.zip › ijms-486730 supplementary final/Figure S3 - S4-TSPYL2 Expression across cancers.pdf]

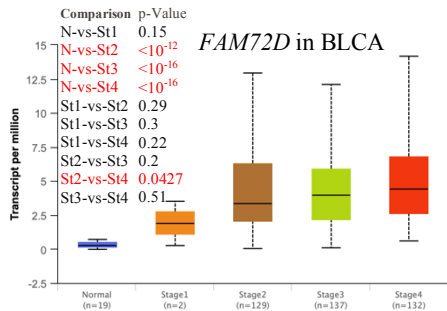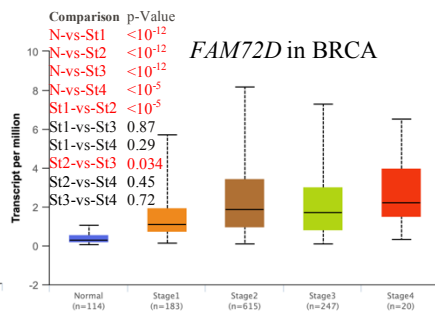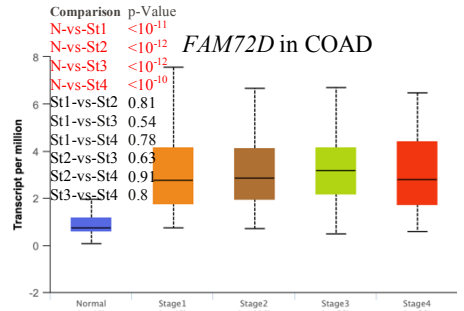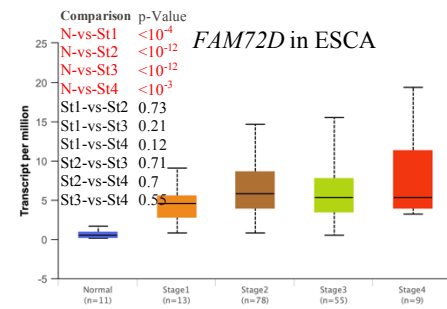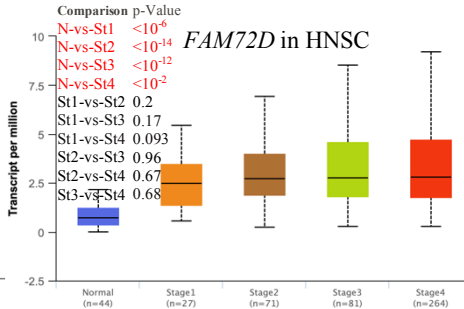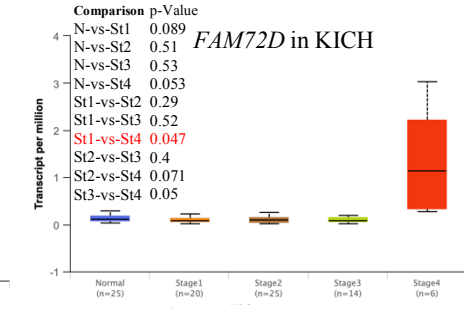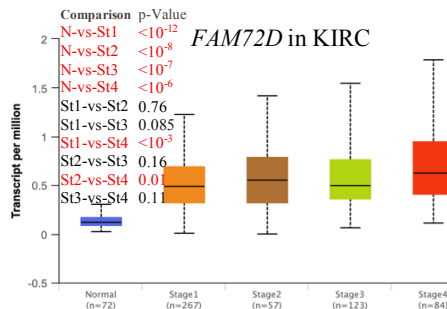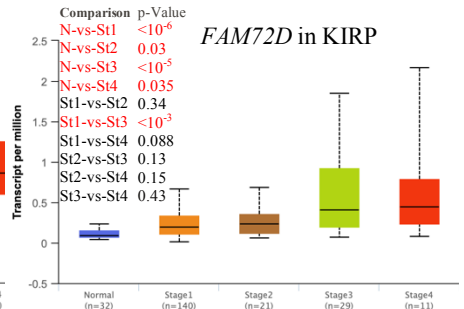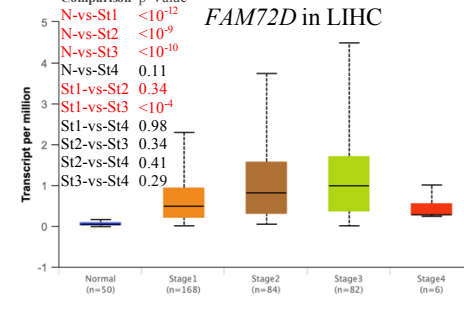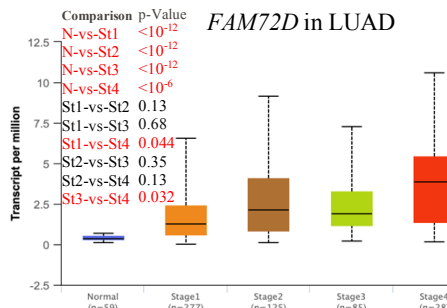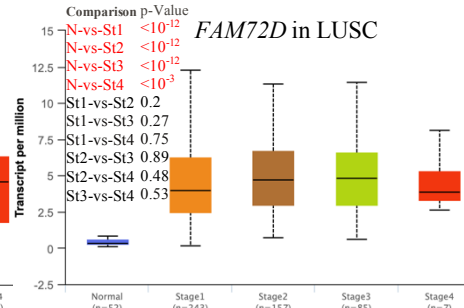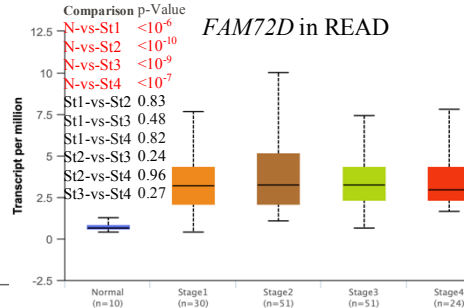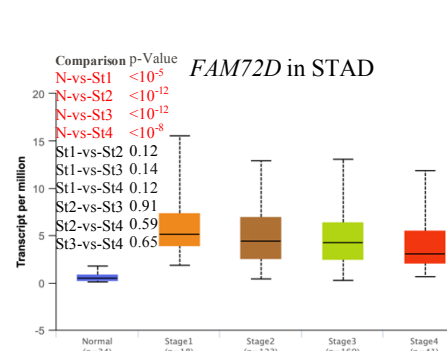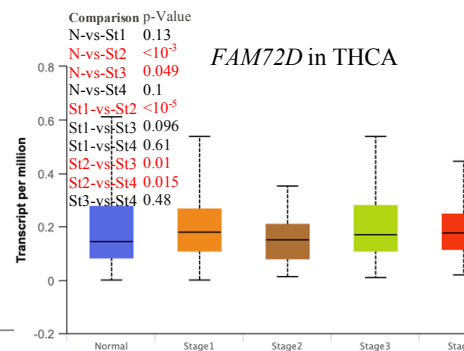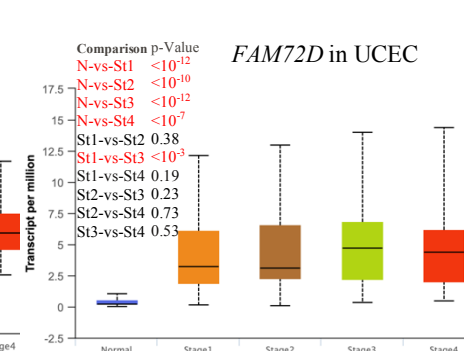

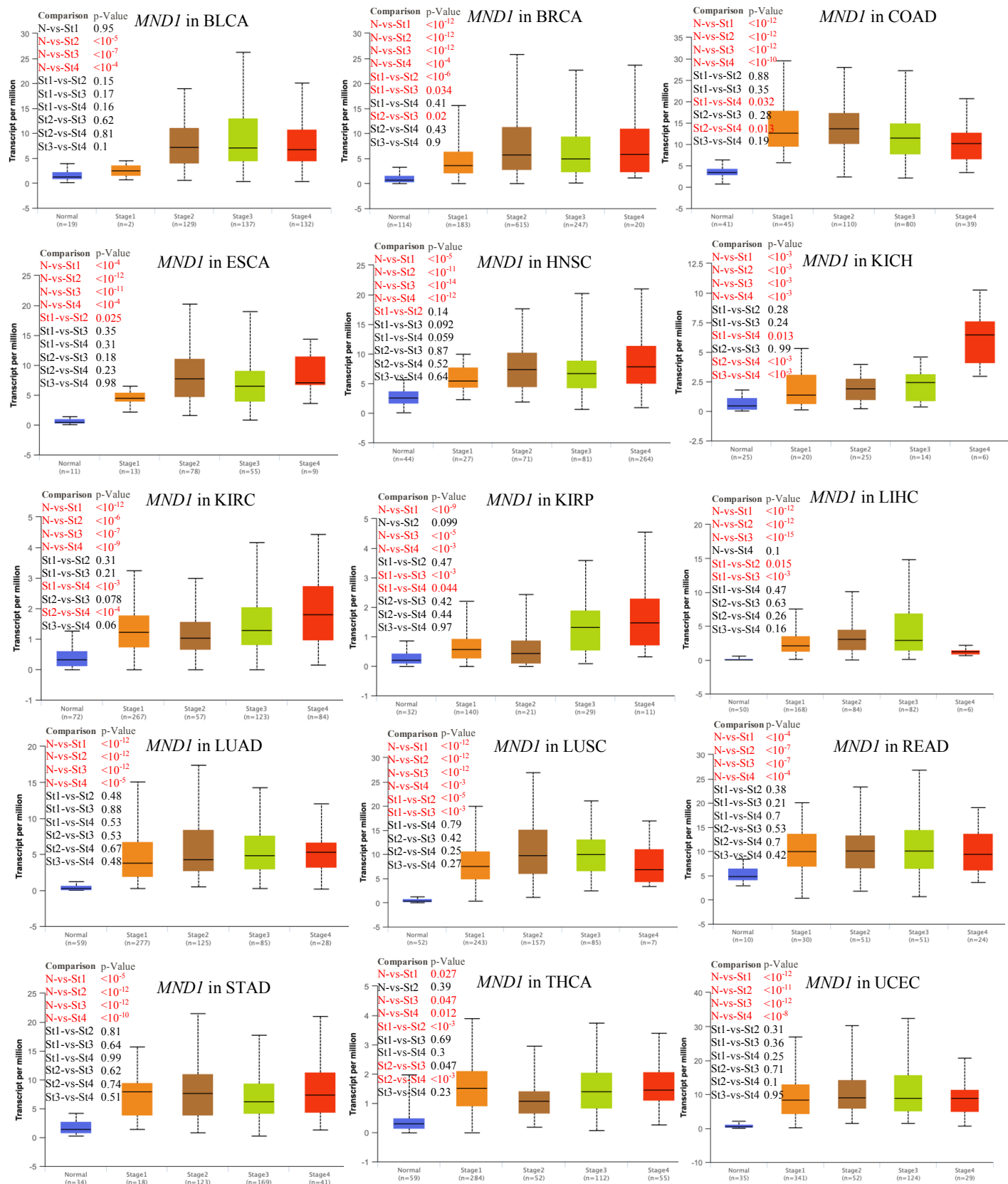

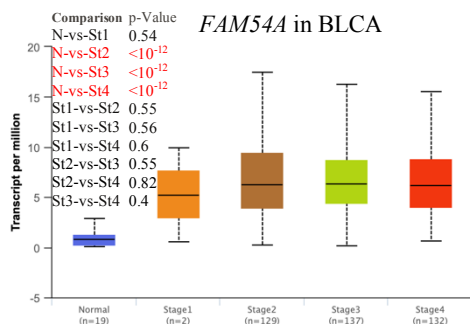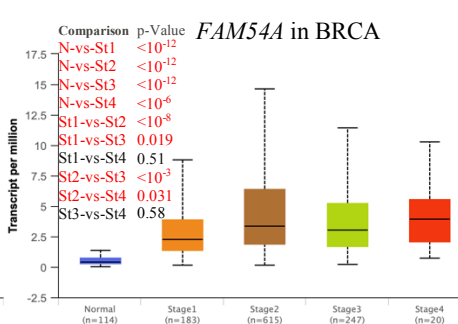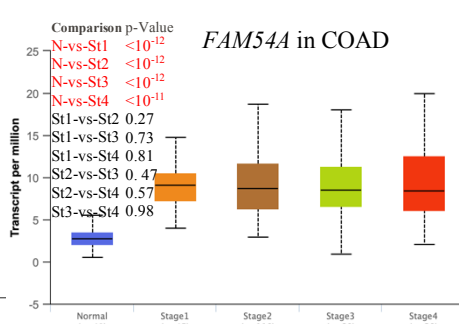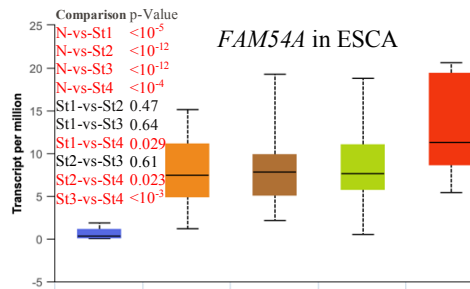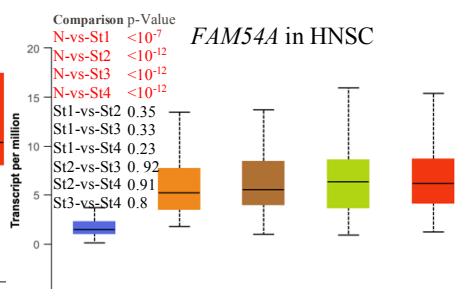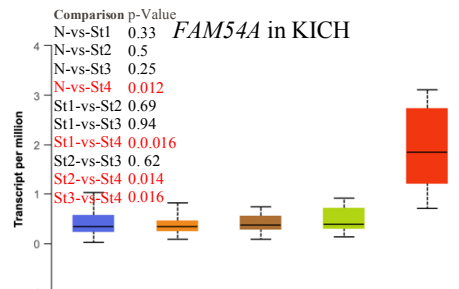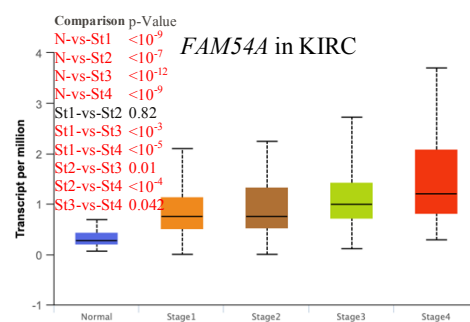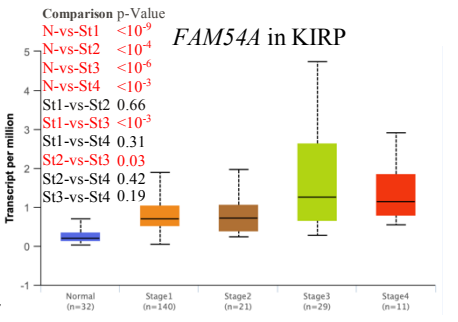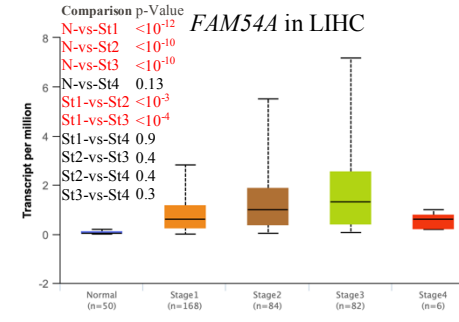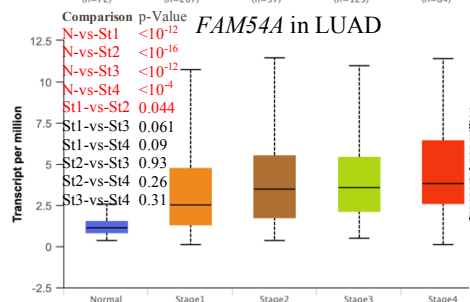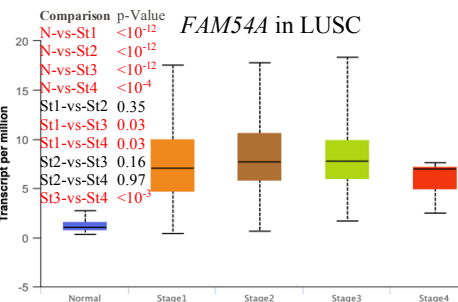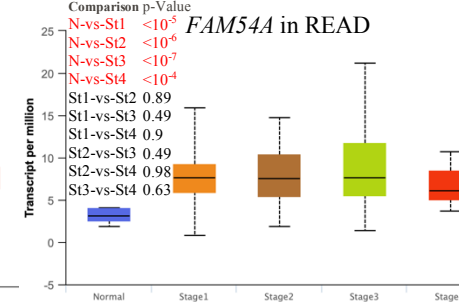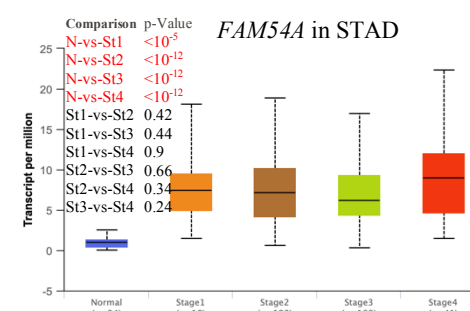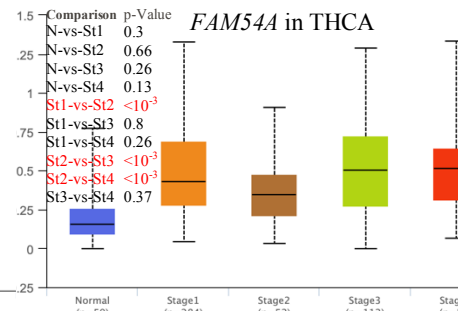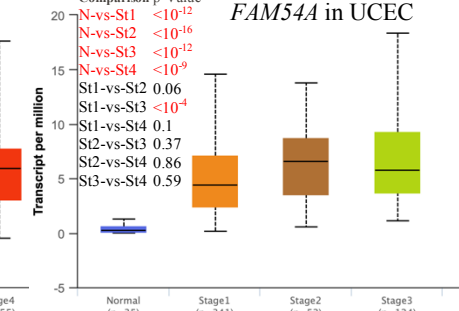

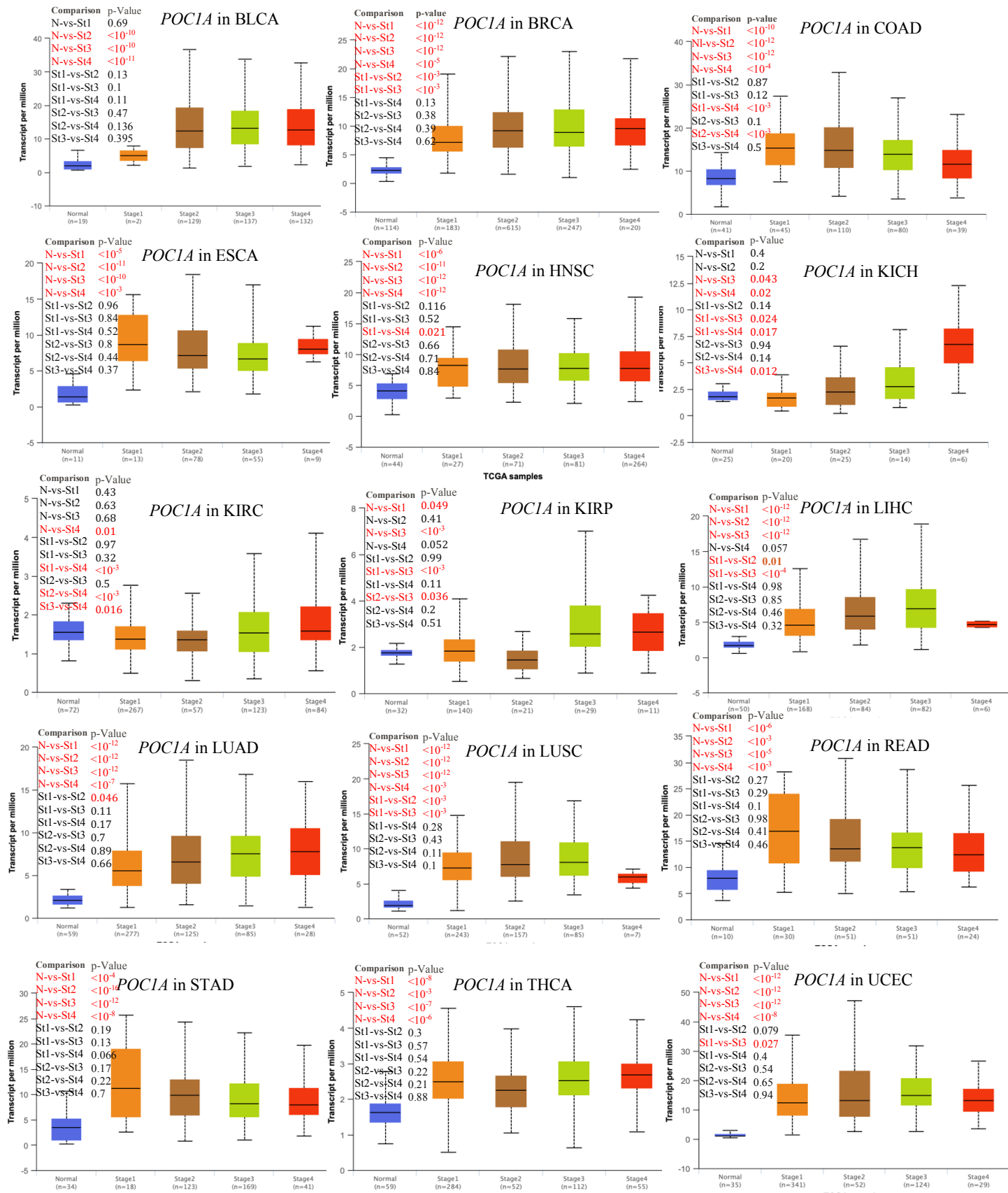

Supplement: Supplementary file 1 [file ijms-20-02228-s001.zip › ijms-486730 supplementary final/Figure S4 - S5. Target genes expression across pathological stages.pdf]
